# Genome-Wide Analysis of Light- and Temperature-Entrained Circadian Transcripts in *Caenorhabditis elegans*

**DOI:** 10.1371/journal.pbio.1000503

**Published:** 2010-10-12

**Authors:** Alexander M. van der Linden, Matthew Beverly, Sebastian Kadener, Joseph Rodriguez, Sara Wasserman, Michael Rosbash, Piali Sengupta

**Affiliations:** 1 Department of Biology and National Center for Behavioral Genomics, Brandeis University, Waltham, Massachusetts, United States of America; 2 Howard Hughes Medical Institute, Brandeis University, Waltham, Massachusetts, United States of America; University of Geneva, Switzerland

## Abstract

Most organisms have an endogenous circadian clock that is synchronized to environmental signals such as light and temperature. Although circadian rhythms have been described in the nematode *Caenorhabditis elegans* at the behavioral level, these rhythms appear to be relatively non-robust. Moreover, in contrast to other animal models, no circadian transcriptional rhythms have been identified. Thus, whether this organism contains a bona fide circadian clock remains an open question. Here we use genome-wide expression profiling experiments to identify light- and temperature-entrained oscillating transcripts in *C. elegans*. These transcripts exhibit rhythmic expression with temperature-compensated 24-h periods. In addition, their expression is sustained under constant conditions, suggesting that they are under circadian regulation. Light and temperature cycles strongly drive gene expression and appear to entrain largely nonoverlapping gene sets. We show that mutations in a cyclic nucleotide-gated channel required for sensory transduction abolish both light- and temperature-entrained gene expression, implying that environmental cues act cell nonautonomously to entrain circadian rhythms. Together, these findings demonstrate circadian-regulated transcriptional rhythms in *C. elegans* and suggest that further analyses in this organism will provide new information about the evolution and function of this biological clock.

## Abstract 2

Transcriptional profiling experiments identify light- and temperature-entrained circadian transcripts in *C. elegans*.

## Author Summary

Daily (circadian) rhythms in behavior and physiology allow organisms to adapt to periodic cues such as light and temperature associated with the rotation of the earth. Subsets of molecular components of the internal clock that drive these rhythms, as well as effector genes for behavioral outputs, also exhibit rhythmic expression in many organisms. While circadian rhythms in behavior have previously been described in the nematode *Caenorhabditis elegans*, no transcriptional rhythms or clock genes have been identified, leaving open the question of the nature of the clock in this organism. Here, we identify light- and temperature-entrained cycling genes in *C. elegans* via genome-wide transcriptional profiling. Transcripts showing circadian regulation (including expression with a 24-h period maintained upon removal of the entraining stimulus) and temperature compensation were identified. Light and temperature appear to entrain independent sets of genes. We also identify large sets of light- or temperature-driven genes. Mutations in a channel gene previously implicated in sensory transduction in a small set of sensory neurons abolish entrainment of gene expression by environmental signals. This work demonstrates the presence of circadian transcriptional rhythms in *C. elegans*, and provides the foundation for future investigations into the underlying mechanisms.

## Introduction

Organisms exhibit daily (circadian) rhythms in behavior and physiology that are attuned to the earth's rotational period. The periods of these intrinsic rhythms are approximately 24 h and are synchronized by external cues or zeitgebers (time givers) such as light and temperature. The molecular composition and regulation of endogenous pacemakers that generate circadian rhythms have been extensively investigated. Both the molecules composing the core clock and the molecular mechanisms of clock function are remarkably divergent across kingdoms, as well as between eukaryotes and prokaryotes, suggesting multiple, independent evolutionary origins for the circadian clock [1]–[6]. However, genes and pathways by which circadian oscillatory rhythms are generated in animals such as *Drosophila* and mammals are strongly conserved [5],[7],[8], implying a single origin of the clock in the metazoan lineage [2]. Thus, it is of interest to identify and characterize the circadian clock in additional animals.

The nematode *Caenorhabditis elegans* is well established as an excellent model organism for the study of development and behavior [9]–[12]. In their soil habitat, worms are subjected to temperature fluctuations corresponding to solar irradiance during the day–night cycles [13],[14]. Moreover, when associated with surface-dwelling animals [15], *C. elegans* is likely to be exposed to daily light–dark cycles. Several studies have previously reported the presence of circadian rhythms in *C. elegans*. In these studies, growth-synchronized populations were entrained to light or temperature cycles, and circadian rhythms in behaviors such as locomotion or responses to osmotic stress were quantified [16]–[20]. Although these experiments demonstrated the presence of rhythms with characteristics of true circadian oscillations, these rhythms were surprisingly variable and non-robust [19]. In addition, while the *C. elegans* genome is predicted to encode homologs of most *Drosophila* and mammalian core clock components, the roles of these genes appear to be largely restricted to development [21]. Thus, whether *C. elegans* exhibits bona fide circadian rhythms remains unclear.

Circadian rhythmicity in behavior and physiology has been proposed to be driven by clock-regulated oscillations in transcription and translation, and/or via post-transcriptional or post-translational modifications [22]–[32]. The expression of core clock genes such as *per* (*period*) and *tim* (*timeless*) as well as many clock output genes cycles at the transcriptional level in a circadian manner in *Drosophila* [23],[24]. Expression profiling experiments have, therefore, provided a powerful approach to identify clock genes as well as clock output genes under circadian transcriptional control [33]–[41]. Since expression profiling is not biased by prior assumptions regarding a specific behavioral or physiological output, transcriptional rhythms may be a robust measure of the presence of a molecular circadian clock.

Here we identify light- and temperature-regulated transcriptional rhythms in *C. elegans* and show that subsets of these transcripts are regulated in a circadian manner. We find that light and temperature also globally drive the expression of many genes, indicating that *C. elegans* exhibits systemic responses to these stimuli. Light- and temperature-entrained transcripts appear to be largely nonoverlapping. We show that conserved clock gene homologs do not exhibit circadian rhythmicity at the mRNA level. We also find that the TAX-2 cyclic nucleotide-gated channel subunit, previously implicated in thermosensory signal transduction and phototransduction [42]–[44] is required to transduce both light and temperature information to the clock(s). These results demonstrate that *C. elegans* has circadian clock(s) that are entrained by light and temperature, and indicate that this model organism can be used to further explore the evolution and function of this critical timekeeping mechanism.

## Results

### Identification of Cycling Transcripts Entrained by Light and Temperature

**Figure 1:**
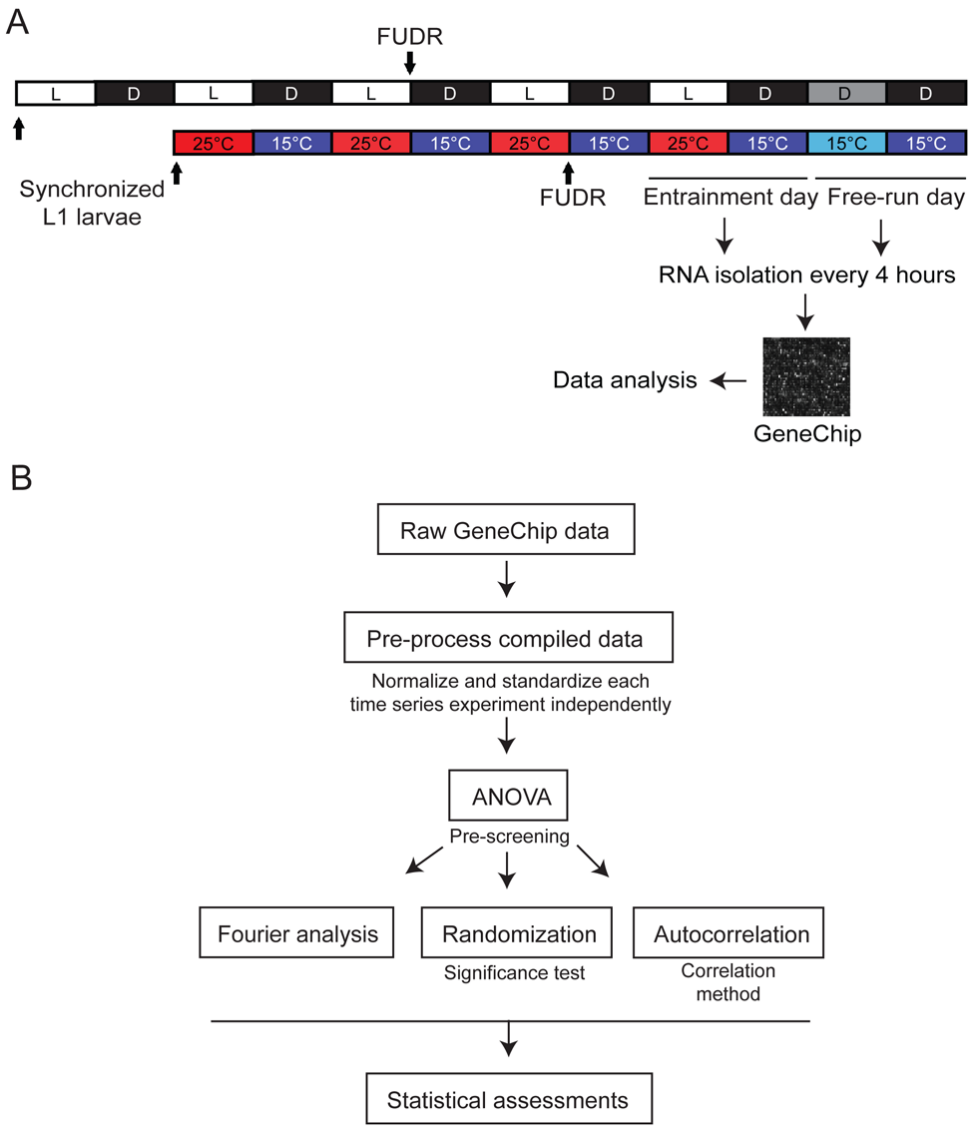
Method for RNA collection and analysis flowchart for detection of cycling transcripts. (A) Populations of growth-synchronized wild-type L1 larvae were entrained for 5 d until adulthood to 12 h∶12 h LD cycles (500–1,000 lux) at a constant temperature of 18°C, or for 4 d to 12 h∶12 h temperature cycles (25°∶15°C [WC]) in DD. RNA was collected every 4 h during the last entrainment and the subsequent free-running days and analyzed via hybridization of Affymetrix GeneChips (see Materials and Methods). L4 larvae were transferred to FUDR-containing plates to inhibit embryonic development. (B) Normalized and standardized expression values from each independent time series experiment were prescreened with ANOVA to identify transcripts that exhibit statistically significant changes in expression over time. Appended datasets from each independent time series experiment were then examined to identify the 24-h spectral power (*F*_24_) score of each transcript [87], and the significance was calculated via comparison with a randomly permuted dataset. The 24-h autocorrelation (AC_24_ score) between time points that were 24 h apart was determined by fitting the six time points collected during the entrainment day to the six time points collected during the free-running day. See Materials and Methods for additional details.

To determine whether cycling transcripts can be identified in *C. elegans* following entrainment to either light–dark or temperature cycles, we performed a genome-wide expression profiling analysis. Growth-synchronized populations of wild-type L1 larvae were entrained until adulthood to either 12 h∶12 h light/dark (LD) cycles at a constant temperature of 18°C, or to 25°C∶15°C (warm/cold [WC]) temperature cycles in constant darkness (DD) (Figure 1A). Following entrainment, animals were maintained under free-running conditions of DD or at a constant temperature of 15°C (constant cold [CC]) (Figure 1A). To exclude non-entrained transcripts present in the embryos of self-fertilizing *C. elegans* hermaphrodites, L4 larvae and adults were grown on plates containing 5-fluoro-2-deoxyuridine (FUDR), which inhibits DNA synthesis and results in embryonic lethality [45]. RNA was collected every 4 h during the last 24 h of entrainment as well as during the first 24 h of free-running conditions (Figure 1A) and hybridized to Affymetrix *C. elegans* Genome Arrays containing 22,500 probe sets representing more than 19,000 predicted *C. elegans* genes.

To identify putative circadian oscillatory transcripts with high confidence, we performed a series of data analyses based on the initial assumption that these rhythms exhibit approximately 24-h periodicity (Figure 1B and Table S1; see Materials and Methods). These algorithms identify cycling transcripts based on consonances in their periods and phases while minimizing the contribution of experimental variations in oscillation amplitude [46]. Similar methods were used in a meta-analysis of circadian expression profiling data from *Drosophila* to identify a set of light- and temperature-entrained circadian transcripts [41],[47]. To validate the algorithms and filters applied by us, we reanalyzed a LD dataset from *Drosophila* (S. K. and M. R., unpublished data) and successfully identified all major core clock genes including *per*, *clk*, *tim*, *cry*, and *vri*, whose expression is known to cycle under these conditions (data not shown). These analyses allowed us to identify transcripts that cycle with a 24-h period in both the light- and temperature-regulated datasets, subsets of which continued to cycle under constant conditions (see below).

### Transcription Rhythms Are Driven, Entrained, and Enriched for 24-h Periods

**Figure 2:**
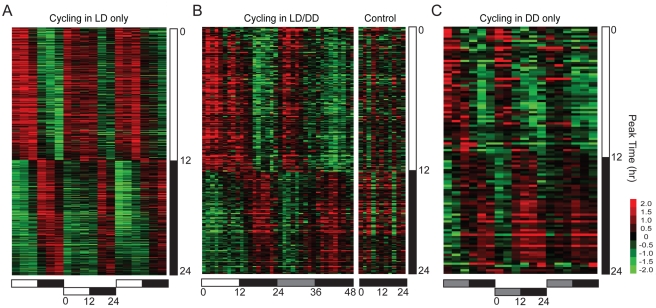
Light-driven and -entrained transcripts. Phase-ordered heat maps showing cycling transcripts upon entrainment to LD cycles. (A) Transcripts that cycle in LD but not in DD. (B) Transcripts that cycle in both LD and DD. (C) Transcripts that cycle in DD but not in LD. Only transcripts that passed all applied filters and thresholds are shown (Table S1). The heat maps in (A) and (C) show appended data from three independent 1-d entrainment time series experiments and three independent 1-d free-running time series experiments, respectively. The heat map in (B) shows the average of three independent 2-d time series experiments. Data from two independent 1-d time series experiments performed in parallel in DD to control for temperature fluctuations are also shown in (B). In all panels, columns represent experimental time points, and rows represent individual transcript profiles. Rows are ordered according to the estimated peak phases of the transcript profiles across the appended or averaged datasets, indicated by the vertical bars to the right of each heat map. Expression values represented by the green-to-red color scale indicate up- or down-regulation relative to the experimental mean expression values indicated in black. Horizontal bars below the heat maps correspond to the LD entrainment protocols used in the time series experiments; white, black, and gray bars in all panels indicate the light, dark, and subjective light phases, respectively.

**Figure 3:**
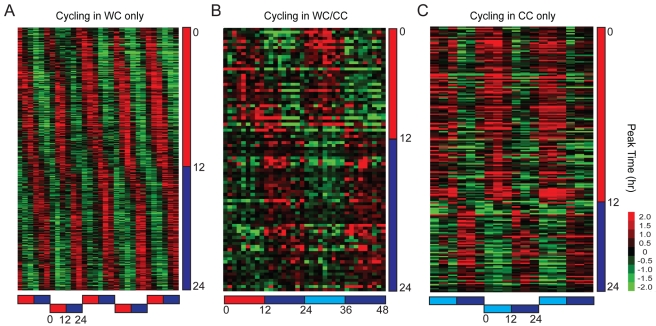
Temperature-driven and -entrained transcripts. Phase-ordered heat maps showing cycling transcripts upon entrainment to temperature cycles (25°C∶15°C). (A) Transcripts that cycle in WC but not in CC. (B) Transcripts that cycle in both WC and CC. (C) Transcripts that cycle in CC but not in WC. Only transcripts that passed all applied filters and thresholds are shown (Table S1). The heat maps in (A) and (C) show appended data from three independent 1-d entrainment time series experiments and three independent 1-d free-running time series experiments, respectively. The heat map in (B) shows the average of three independent 2-d time series experiments. Data are presented as in Figure 2. Horizontal bars below the heat maps correspond to the WC entrainment protocols used in the time series experiments; red, blue, and light blue bars in all panels indicate the warm, cold, and subjective warm phases, respectively.

Entrainment via light or temperature cycles may drive as well as entrain transcriptional rhythms [47]. Stimulus-driven but clock-independent transcripts are expected to cycle during entrainment but not during free-running conditions, as observed in the first category of cycling transcripts (LD only or WC only) (Figures 2A and 3A). The second category included transcripts that cycled during both entrainment and free-running conditions (LD/DD or WC/CC) (Figures 2B and 3B), suggesting that expression of these genes may be under circadian control [47]. We also identified a third category of transcripts whose expression cycled during the free-running but not during the entrainment conditions (DD only or CC only) (Figures 2C and 3C). This category may include genes whose expression is also under circadian control, but whose cycling is suppressed or masked during entrainment conditions. A similar category of transcripts was previously noted in genome-wide studies of light-regulated transcriptional rhythms in *Drosophila* [36].

**Figure 4:**
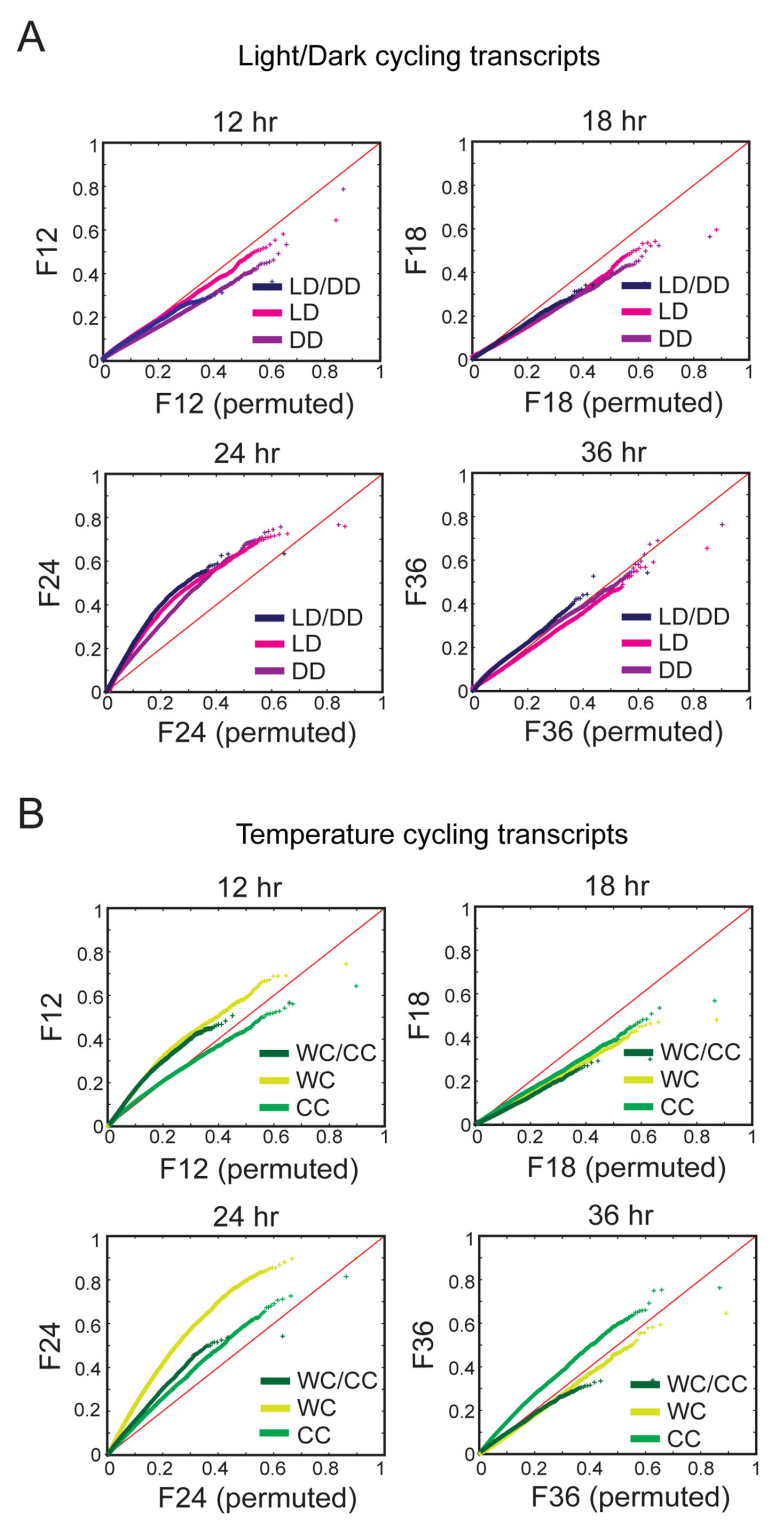
24-h rhythms are enriched in the light- and temperature-entrained datasets. QQ plots of Fourier scores at four different periods (12, 18, 24, and 36 h) are shown from LD-entrained (A) and temperature-entrained (B) datasets subdivided into categories as indicated in Figures 2 and 3. The Fourier scores of each transcript (with log_2_-transformed expression value >10) are graphed against the randomly permuted 95^th^ quantile Fourier scores. Enrichment of the experimental period is shown as an upward departure from the diagonal (red line), while depletion is shown as a downward departure.

We investigated whether a 24-h circadian period is enriched in our datasets by comparing the distribution of Fourier scores with different periods with randomly permuted Fourier scores in quantile–quantile (QQ) plots. All LD datasets (LD only, LD/DD, and DD only) were significantly enriched for 24-h periods, whereas distributions of 12-h, 18-h, and 36-h period data were indistinguishable from those of randomly permuted data (Figure 4A). Although 24-h periods were also similarly enriched in the WC datasets (WC only, WC/CC, and CC only), we found lower, but significant enrichment of 12-h and 36-h periods in the WC-only and WC/CC datasets and in the CC-only datasets, respectively (Figure 4B). These results indicate robust circadian regulation of transcripts, with infradian and ultradian rhythms also observable in the temperature-regulated datasets. This pattern is similar to higher order harmonics of circadian gene expression previously identified in mouse liver [48].

### Temperature Cycles Drive the Expression of a Large Set of Genes Implicated in Metabolic Processes

We next further analyzed the light- and temperature-driven transcripts (LD-only and WC-only datasets, respectively). The number of putative temperature-driven transcripts was significantly higher than the number of light-driven transcripts: 1,817 WC-driven compared to 775 LD-driven transcripts (Figures 2A and 3A). These comprise approximately 9% and 4% of all *C. elegans* genes, respectively, underscoring the critical roles of temperature and light in regulating *C. elegans* physiology.

**Figure 5:**
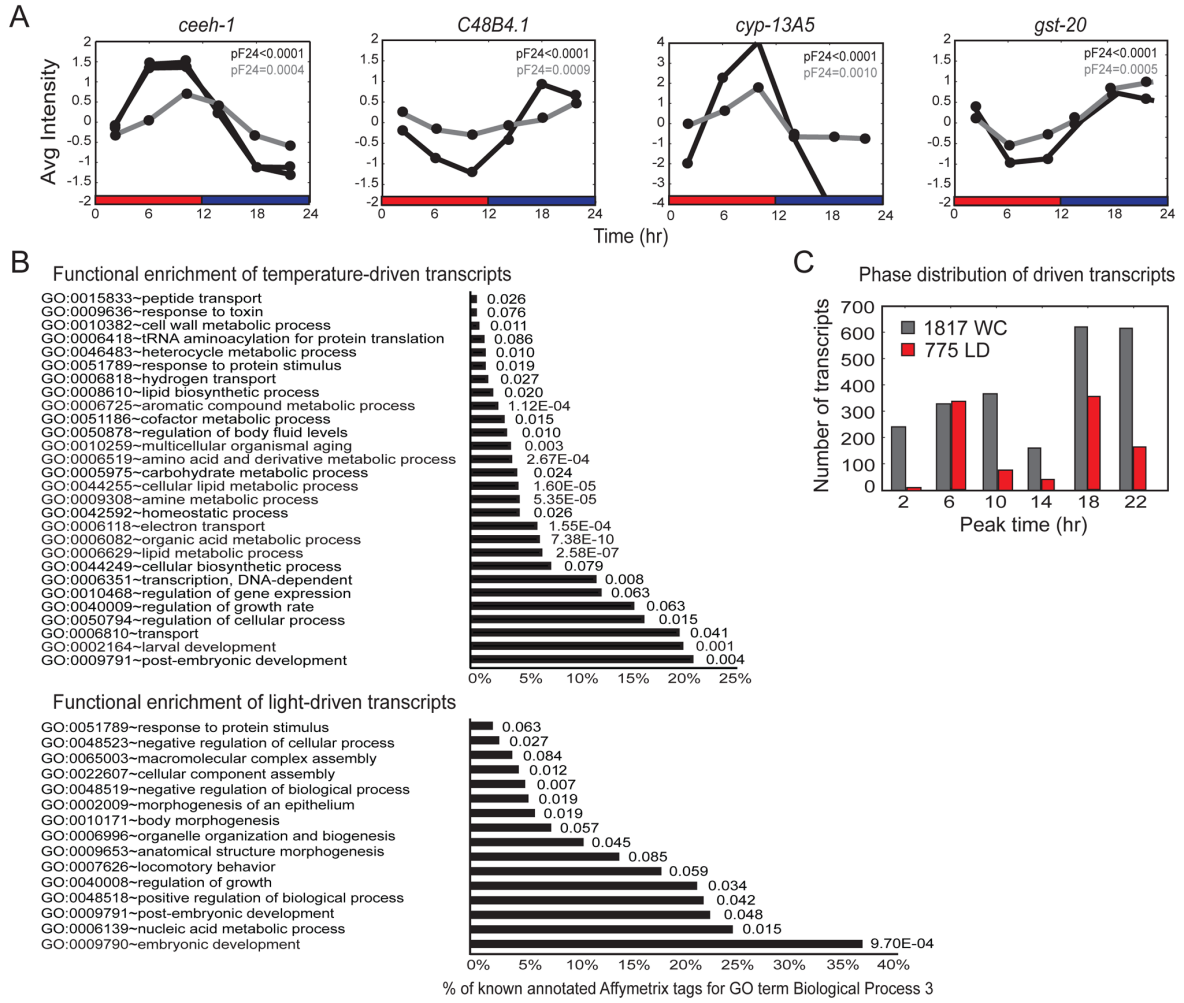
Analyses of light- and temperature-driven transcripts. (A) Comparison of qRT-PCR (gray) and microarray expression array (black) data for four arbitrarily selected WC-driven transcripts. Additional transcripts are shown in Figure S1. The probability of significance of circadian cycling (*pF*_24_) as compared to a randomized dataset was calculated by appending each independent microarray or qRT-PCR time course experiment. Bars below the graphs denote the entrainment protocols, with red and blue bars indicating the warm (25°C) and cold (15°C) phases, respectively. Data shown are an average of three biologically independent replicates per time point for the microarray data, and two biologically independent replicates per time point for the qRT-PCR data. Data from two probe sets on the GeneChips for *ceeh-1* are shown. (B) Functional enrichment of LD- and WC-driven transcripts. Transcripts are grouped by the Biological Process 3 GO term and analyzed for enrichment relative to all transcripts present on the GeneChip (see Materials and Methods). *p*-values for enrichment for each group are indicated. A complete list is provided in Table S2. (C) Histogram showing the estimated peak phases of the temperature- or light-driven transcripts. The peak phases are organized into 4-h phase groups.

We verified rhythmic expression of selected genes by performing quantitative reverse transcription PCR (qRT-PCR) analyses. Each of the 12 examined genes from the WC-only dataset exhibited significant cycling, with phases consistent with those identified in the transcriptional profiling experiments (Figures 5A and S1A). Expression was arrhythmic in the absence of imposed temperature cycles (Figure S1B). These driven genes are predicted to be implicated in diverse biological pathways (Figure 5B and Table S2). Based on the biological process Gene Ontology (GO) terms [49], categories of metabolism and electron transport were markedly enriched in the WC-only dataset (Figure 5B and Table S2). For instance, the expression of 18 genes predicted to encode cytochrome P450 enzymes was driven by WC cycles, whereas only two cytochrome P450 genes were present in the LD-driven dataset (Table S2). This suggests that temperature, but not light, modulates the expression of genes implicated in global regulation of metabolism or physiology. Genes associated with the GO term embryonic development were enriched in the LD-only-driven dataset (Figure 5B and Table S2).

We ordered the transcripts in each dataset by the average phase of peak expression (Figure 5C). We found that the peak phases were around the middle of the thermo–light or the cryo–dark phases. For LD transcripts, there were similar numbers of transcripts at these two phases, whereas more WC transcripts peaked during the cryophase than in the thermophase (Figure 5C), similar to previous observations in *Drosophila* [47]. Together, these results indicate that temperature and light cycles drive global changes in gene expression in *C. elegans*.

### Putative Circadian Transcripts Represent Diverse Biological Functions

**Figure 6:**
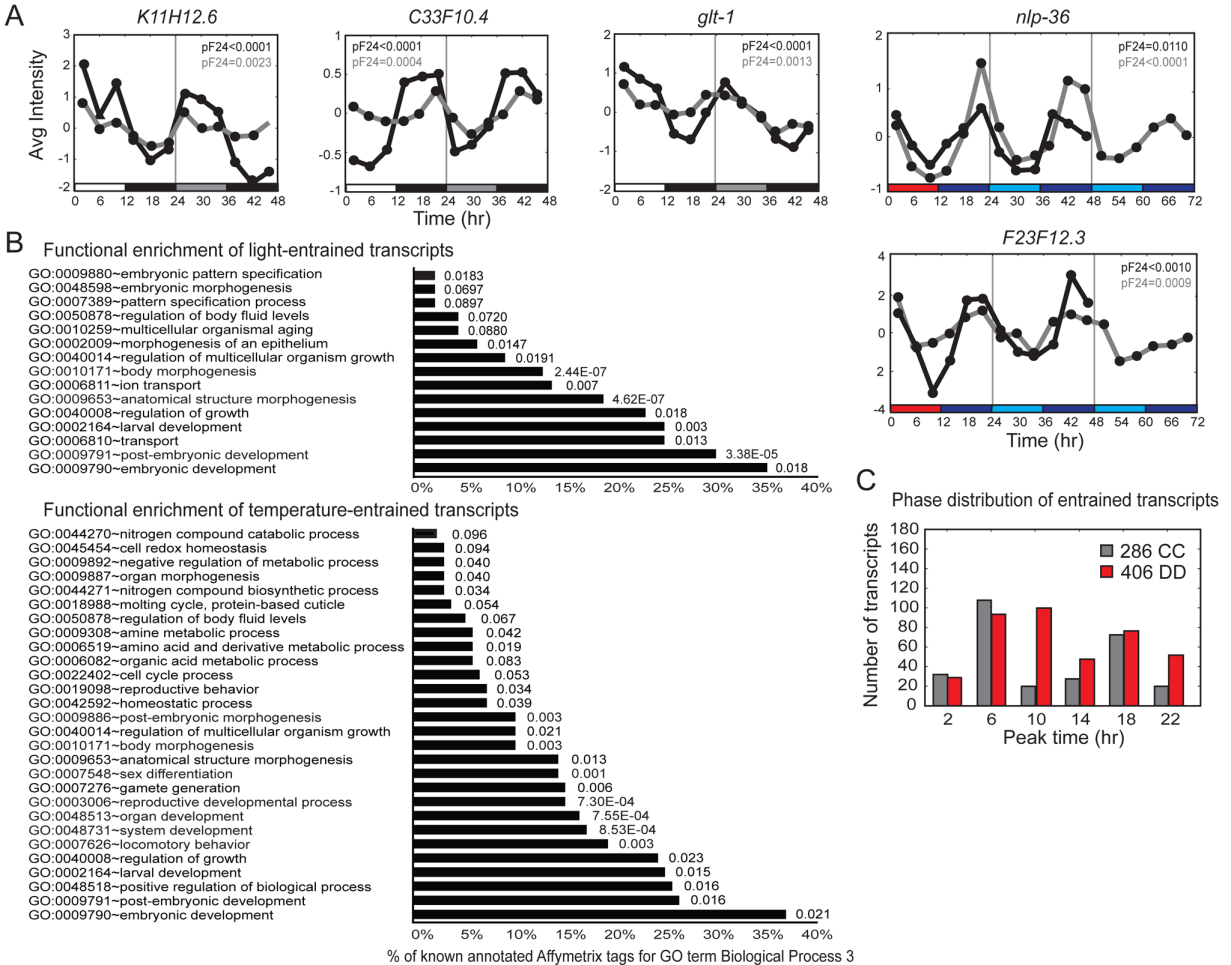
Analyses of light- and temperature-entrained circadian transcripts. (A) Comparison of qRT-PCR (gray) and microarray expression array (black) data of randomly selected LD- and WC-entrained transcripts. Additional transcripts are shown in Figure S2. Bars below the graphs denote the entrainment protocols, with white, black, and gray bars indicating the light, dark, and subjective light phases, respectively, and with red, blue, and light blue indicating the warm (25°C), cold (15°C), and subjective warm phases, respectively. Data shown are an average of three biologically independent replicates per time point for the microarray data and two biologically independent replicates per time point for the qRT-PCR data. (B) Functional enrichment of light- and temperature-entrained circadian transcripts, as shown in Figure 5B. A complete list is provided in Table S3. (C) Histogram showing the estimated peak phases of the temperature- or light-entrained transcripts. The peak phases are organized into 4-h phase groups.

In total, 406 and 286 transcripts were found to show oscillatory expression during the DD and CC free-running conditions, respectively (see Materials and Methods). The subset of these transcripts that cycle both during the LD and WC entrainment conditions and during free-running conditions may be driven as well as entrained. We verified the cycling of selected transcripts by performing qRT-PCR (Figures 6A and S2A). Their phases were similar to those defined via expression profiling, and cycling was similarly dependent on prior entrainment (Figure S2B). Following entrainment, circadian genes sustain cycling in constant conditions. albeit with a considerably diminished amplitude in some systems over extended time periods. We therefore examined the expression of two temperature-entrained genes (*nlp-36* and *F23F12.3*) through 2 d of free-running conditions. Both transcripts continued to exhibit rhythmic transcription during the second free-running day, with highly significant 24-h spectral power values (Figure 6A).

Genes associated with the GO term categories of post-embryonic morphogenesis and development were significantly enriched in both circadian datasets, in contrast to the light- and temperature-driven transcripts (Figure 6B and Table S3). We noted that genes implicated in reproductive development, and to a lesser extent locomotion, were enriched in the temperature-entrained dataset, suggesting that processes such as mating, fertilization, egg-laying, and locomotor behaviors may be under circadian control.

To determine the phase distribution of the light- and temperature-entrained transcripts, we grouped the cycling transcripts identified in the DD- and CC-entrained datasets in 4-h clusters according to their phases of peak expression. The peak phases of circadian transcripts in the light-entrained dataset occurred at around the middle of the light and dark phases, although with a relatively broad distribution (Figure 6C). The phase distribution for temperature-entrained transcripts exhibited a narrower peak in the middle of the thermophase, with another smaller peak in the middle of the cryophase. Although these distributions were not identical to those observed in the driven data, there were some similarities: they both peaked in the middle of the light (warm) and dark (cold) phases and with similar numbers of LD-entrained or -driven transcripts in the two peaks. However, more WC-driven transcripts peaked in the cryophase whereas more WC-entrained transcripts peaked in the thermophase (compare Figures 5C and 6C).

### The Periods of Putative Circadian Transcripts Are Temperature-Compensated, and Their Phases Are Altered by *T*-Cycles

**Figure 7:**
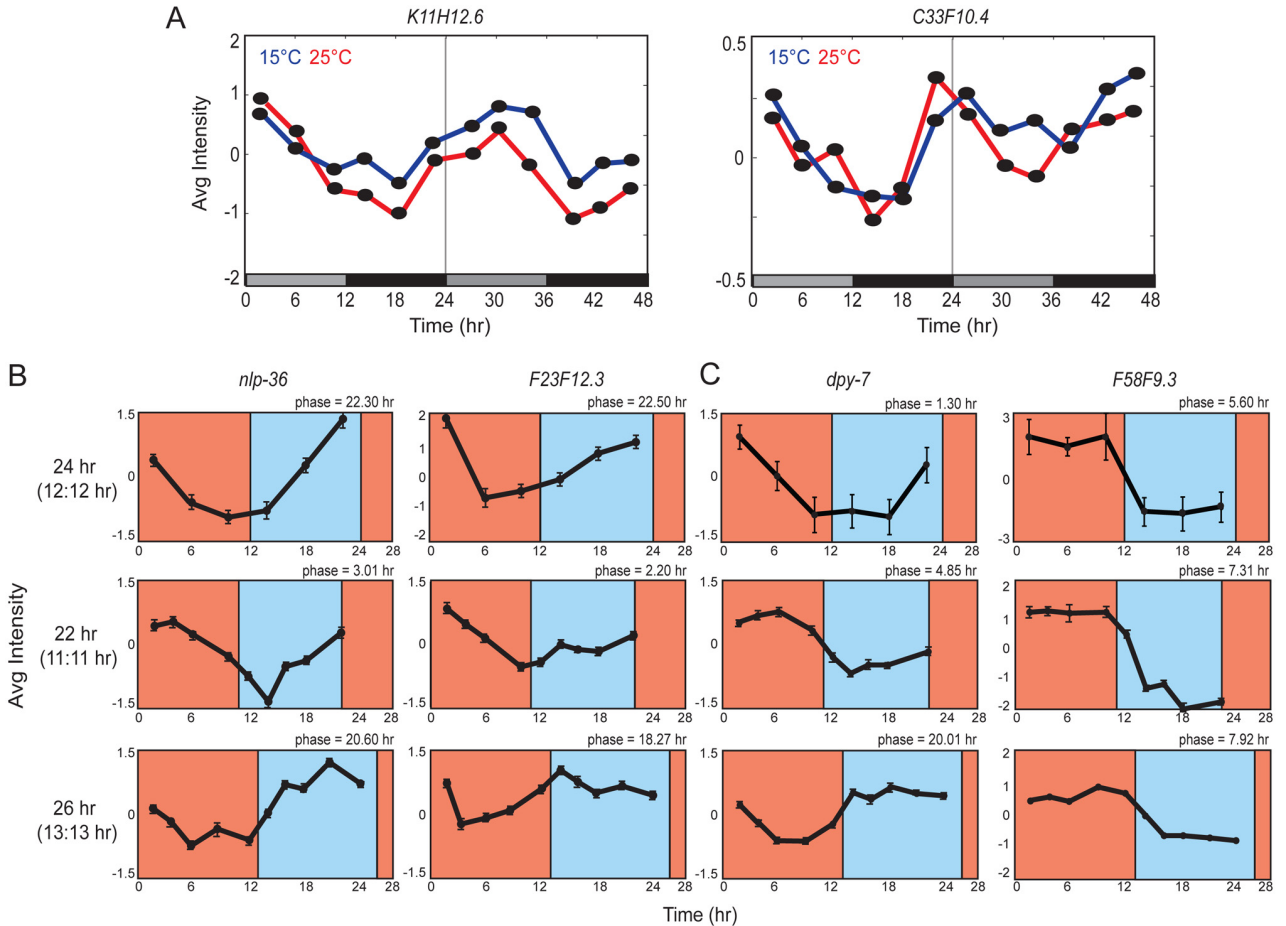
Temperature compensation and phase shifts of entrained transcripts. (A) Cycling of two light-entrained transcripts during the first 2 d of free-running conditions at 15°C (blue line) or 25°C (red line) quantified via qRT-PCR. Calculated periods are as follows: *K11H12.6*, 23.5±0.40 h at 15°C and 23.90±0.70 at 25°C; *C33F10.4*, 23.35±0.25 h at 15°C and 23.45±0.25 h at 25°C. Fourier scores and associated probabilities are shown in Table S4. The bars below the graphs denote the entrainment protocol, with black and gray bars indicating the dark and subjective light phases, respectively. Data shown are from two biological replicates. (B and C) Phase shifts upon entrainment to different *T*-cycles. Shown is the expression of two genes each from the WC-entrained (B) and WC-driven (C) datasets as quantified from GeneChip or qRT-PCR data. RNA was collected on the fourth entrainment day. The expression of additional driven transcripts is shown in Figure S3. Data shown are the average of two technical replicates from one biological experiment.

A hallmark of circadian rhythms is temperature compensation, i.e., the circadian period is almost independent of temperature [50],[51]. To compare periods of two light-entrained transcripts upon growth at 15°C and 25°C, we examined transcript levels via qRT-PCR. The calculated periods and significant periodicities for each transcript were nearly identical at both temperatures (Figure 7A and Table S4), indicating temperature compensation.

Another characteristic of circadian rhythms is the maintenance of a phase relationship with the period (*T*) of the zeitgeber [52]–[55]. Typically, phases are delayed relative to those in 24-h *T*-cycles upon entrainment to *T*-cycles shorter than 24 h, and, conversely, phases are advanced when entrained to *T*-cycles longer than 24 h. We entrained animals to either 11 h∶11 h or 13 h∶13 h WC cycles and examined the phases of two candidate circadian transcripts by qRT-PCR. The phases of both transcripts were delayed in response to 11 h∶11 h *T*-cycles and advanced in response to 13 h∶13 h *T*-cycles (Figure 7B), further suggesting that these transcripts are under circadian regulation.

### Light–Dark and Temperature Cycles Entrain Independent Gene Sets

**Figure 8:**
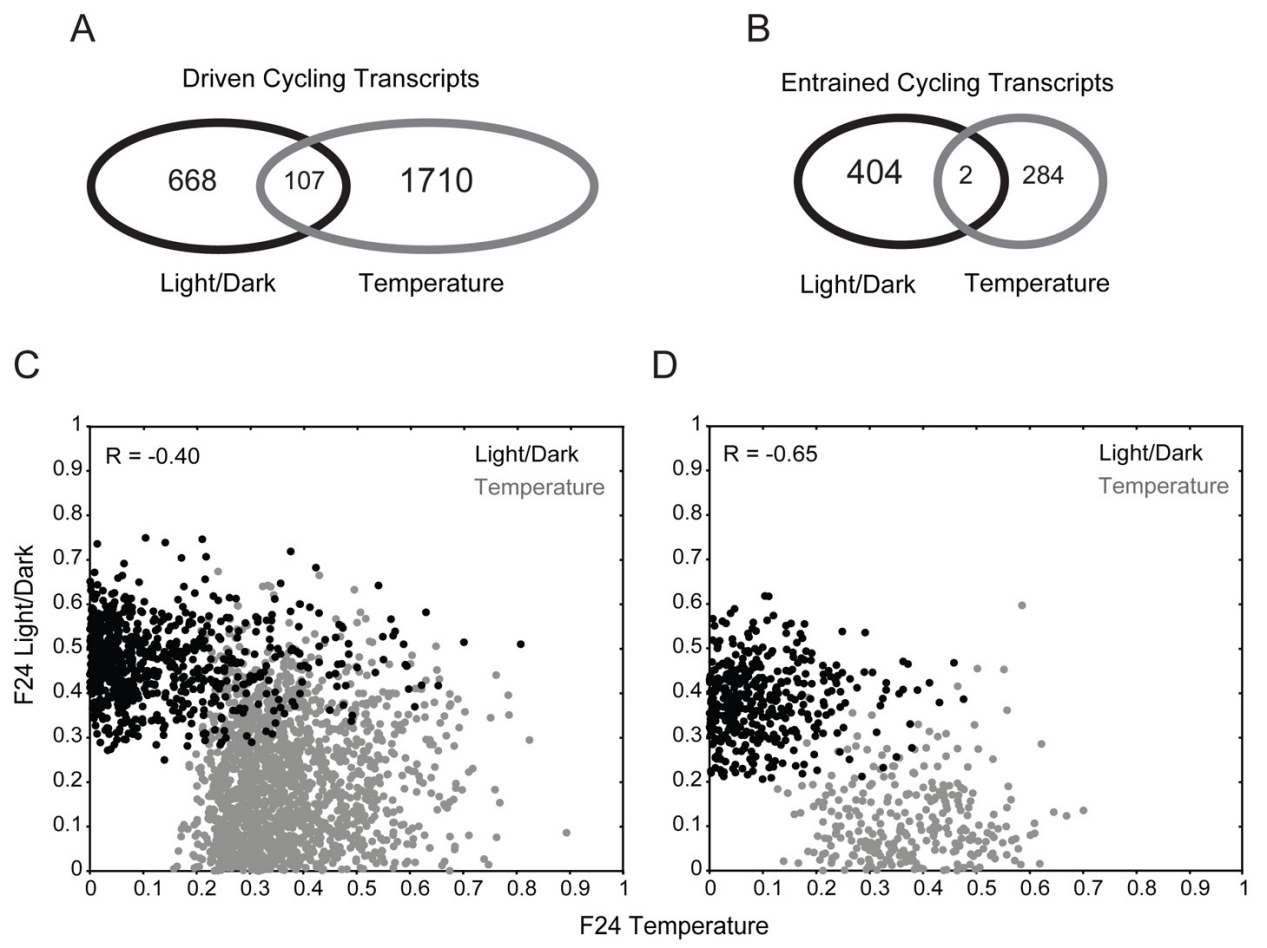
Temperature and light entrain and drive the expression of distinct genes. (A and B) Overlap between light- and temperature-driven cycling transcripts (A), and light- and temperature-entrained cycling transcripts (B). (C and D) Scatter plots of the *F*_24_ scores of each transcript in the driven (C) or the entrained (D) datasets. *F*_24_ scores for light–dark and temperature regulation are shown in black and gray, respectively.

We next compared the two pairs of datasets driven and entrained by light and temperature cycles, to determine the extent to which the two zeitgebers regulate similar sets of genes. A direct comparison of the light- and temperature-driven datasets identified 107 transcripts in common, representing 106 genes (Figure 8A). In the entrained sets, there were only two genes in common (Figure 8B). One of these genes is predicted to be a pseudogene (*Y102A5C.6*), and the second gene (*swd-3.3*) is predicted to encode a homolog of the WDR5 member of the histone methyltransferase complex. Interestingly, this protein is known to associate with mammalian PER1 and regulate its functions [56].

Since the extent of overlap between the datasets is highly dependent on the applied filters and thresholds, we also examined the correlation between the *F*_24_ scores for light–dark and temperature regulation for each transcript in the driven and entrained datasets. We found strong negative correlation in both cases (Figure 8C and 8D), further implying that light–dark and temperature cycles regulate independent sets of genes.

We considered the possibility that the lack of overlap between the entrained datasets may be due to the presence of additional circadian transcripts in the driven datasets. The expression of these transcripts may dampen quickly in constant conditions, precluding their inclusion in the entrained datasets. These transcripts may be distinguished from truly driven genes by being phase-shifted in response to *T*-cycles that are longer or shorter than 24 h. To address this hypothesis, we subjected seven arbitrarily selected transcripts from the WC-only-driven datasets to 11 h∶11 h or 13 h∶13 h *T*-cycles. Although the phases of six of seven of these transcripts did not exhibit the expected shifts, the phase of *dpy-7* was advanced or delayed similarly to the examined entrained transcripts (Figures 7C and S3). This observation suggests that the driven datasets may include additional entrained genes, some of which may be entrained by both light and temperature.

### Clock Gene Homologs in *C. elegans* Do Not Exhibit Rhythmic Expression in Whole Animal Profiling Experiments

Although a subset of core clock genes, such as the *doubletime* casein kinase gene, are expressed constitutively [57], genes such as *per* or *tim* exhibit circadian transcription in *Drosophila* [23]–[26]. The *C. elegans* genome is predicted to encode homologs of core clock genes implicated in circadian regulation in *Drosophila* and mammals (www.wormbase.org) [21], and animals mutant for the *per* homolog *lin-42* exhibit an increased circadian period in locomotor behavior upon entrainment to light or temperature cycles [19]. However, upon entrainment by either light or temperature zeitgebers, no significant rhythmicity was found for the expression of the *lin-42 (per)*, *tim-1 (tim)*, *aha-1 (clk/cyc)*, or *atf-2 (vri)* genes (Table S5). We also did not observe changes in *green fluorescent protein (gfp)* expression upon temperature entrainment of wild-type animals transgenic for *atf-2*p*::gfp* and *aha-1*p*::gfp* fusion genes (data not shown). It remains possible, however, that clock gene homologs cycle in a small subset of cells or tissues in *C. elegans*, and/or that these genes exhibit rhythmic expression at the post-transcriptional level.

### Cycling Expression of the *nlp-36* Neuropeptide Gene Can Be Monitored In Vivo

**Figure 9:**
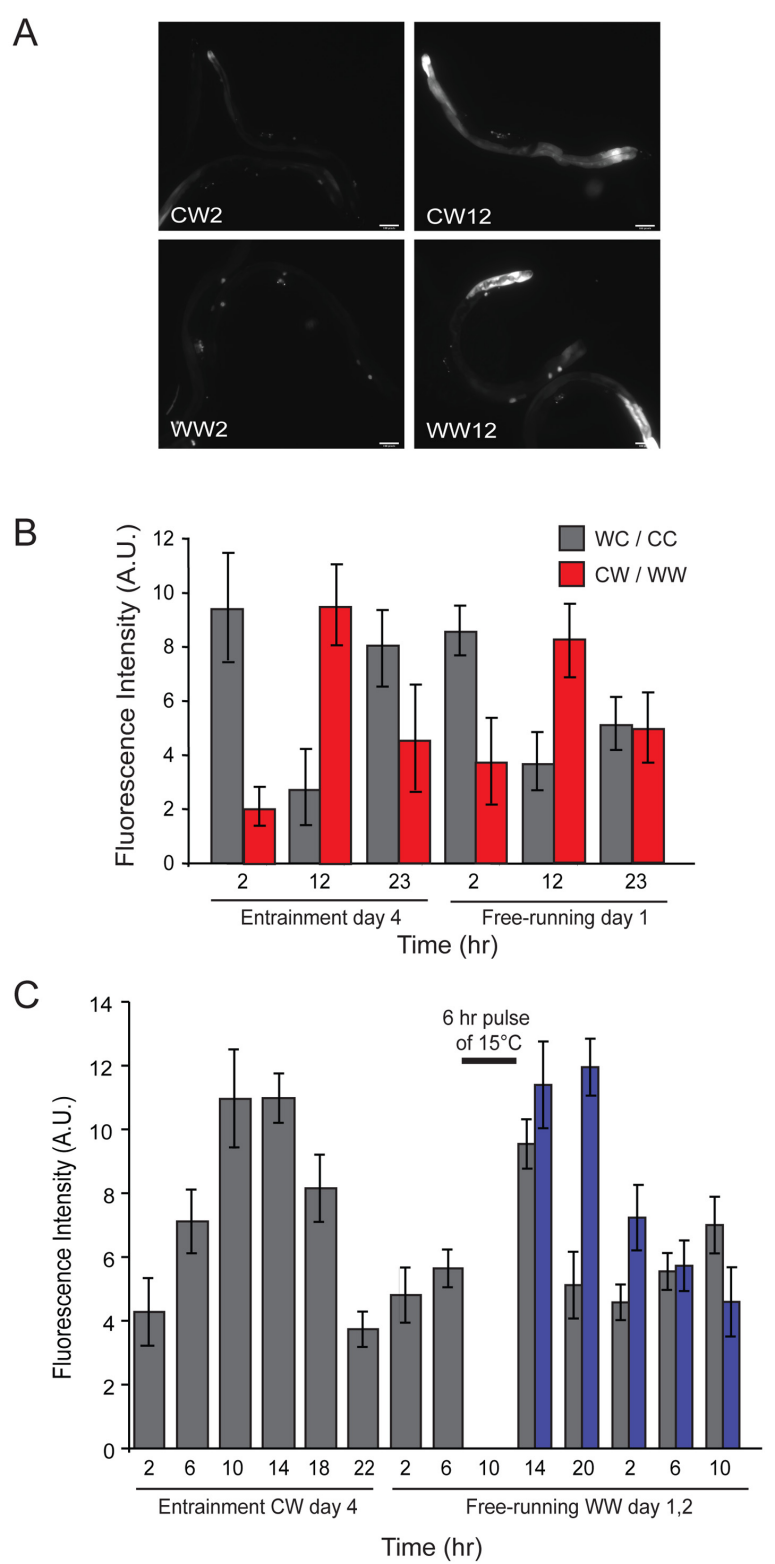
Expression of an *nlp-36*p*::gfp* fusion gene is entrained by temperature cycles. (A) *gfp* expression in transgenic animals carrying stably integrated copies of an *nlp-36*p*::gfp* fusion gene during temperature entrainment (CW) and free-running (WW) conditions. Adult animals were examined under 100× magnification. (B) Quantification of intestinal *nlp-36*p::*gfp* fluorescence intensity (in arbitrary units [A.U.]) at the indicated time points during entrainment or free-running days. Wild-type transgenic animals were entrained to either WC/CC (gray bars) or CW/WW (red bars) cycles. Error bars indicate the s.e.m. Data shown are from two independent experiments with 15–20 animals at each time point. (C) *nlp-36*p*::gfp*-expressing transgenic animals were subjected to CW entrainment cycles and moved to WW conditions. After 6 h at WW, a subset of animals were subjected to a pulse of 15°C temperature for 6 h and returned to WW conditions. Intestinal fluorescence intensities in animals subjected or not subjected to the cold pulse are indicated in blue and gray, respectively. Error bars indicate the standard deviation. *n* = 15–20 animals each at each time point.

To monitor the rhythmic expression of candidate entrained genes in vivo in *C. elegans*, we established a real-time *gfp*-based reporter system. Expression of the *nlp-36* neuropeptide gene exhibits strong temperature-entrained rhythmic expression (Figure 6A and Table S3). We generated strains carrying stably integrated transgenes in which the *nlp-36* promoter drives expression of the *gfp* reporter gene fused to sequences encoding a rapidly turning over PEST domain [58]. The *nlp-36*p*::gfp* fusion gene was expressed in the intestine and additional unidentified cells in the head and tail in adult animals (Figure 9A). We subjected transgenic animals to either cold/warm (CW) or WC cycles and quantified GFP fluorescence levels in the intestine at defined time points.

**Figure 10:**
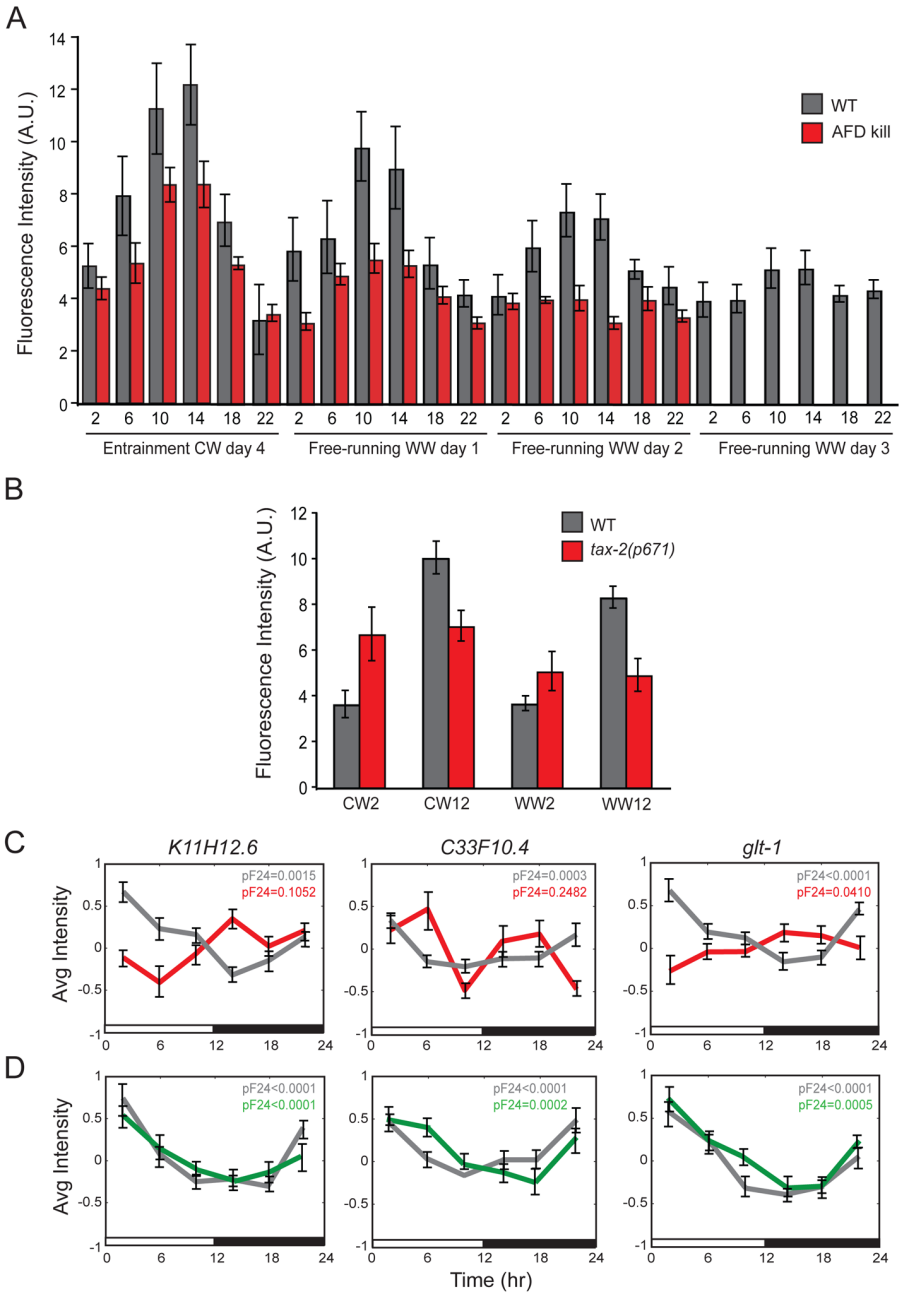
Rhythmic expression of *nlp-36*p*::gfp* is abolished in *tax-2* mutants. (A) Quantification of intestinal *nlp-36*p*::gfp* expression levels in wild-type (gray bars) or AFD-killed animals (red bars) at defined time points during entrainment (CW) followed by 3 d of free-running (WW) conditions. Error bars indicate the s.e.m. Data shown are from two independent experiments of 20 animals each. (B) Fluorescence intensities in wild-type animals (gray bars) or *tax-2* mutant animals (red bars) carrying the *nlp-36*p*::gfp* transgene entrained to temperature cycles (CW/WW). Error bars indicate the s.e.m. Data shown are from two independent experiments. (C) Cycling of selected light-entrained transcripts in wild-type animals (gray) and in *tax-2(p671)* mutants (red) quantified via qRT-PCR. The probabilities of circadian cycling (*pF*_24_) by appending each independent qPCR time course experiment are indicated. (D) Cycling of selected light-entrained transcripts in wild-type animals (gray) and in *lite-1(ce314)* mutants (green) quantified via qRT-PCR. The probabilities of circadian cycling (*pF*_24_) are indicated. The bars below the graphs in (C) and (D) denote the entrainment protocol, with white and black bars indicating the light and dark phases, respectively. Data shown are from two independent biological replicates per time point. Error bars indicate the s.e.m. *n* = 15–20 animals each.

*nlp-36*p*::gfp* expression cycled robustly both during entrainment and under free-running conditions, with the expected opposite relative phases in response to CW or WC cycles (Figure 9B). Moreover, cycling persisted for at least two subsequent days in free-running conditions, albeit with dampened amplitude (Figure 10A). Consistent with the absence of *nlp-36* in the set of light-entrained transcripts, we did not observe cycling of *nlp-36*p*::gfp* with light entrainment (Figure S4).

To determine whether the phase of cycling *nlp-36*p*::gfp* expression could be reset upon exposure to a different entraining zeitgeber schedule, we entrained *nlp-36*p*::gfp*-expressing transgenic animals to 12 h∶12 h CW cycles for 4 d. The animals were then shifted to 25°C conditions for 6 h, subjected to cold temperatures (15°C) for 6 h, and returned to 25°C. Quantification of intestinal *gfp* expression levels showed that administration of this temperature pulse resulted in a phase delay (Figure 9C), indicating that *nlp-36* cycling is regulated by a circadian clock.

### Light and Temperature Entrainment of *nlp-36* Expression Requires the *tax-2* Cyclic Nucleotide-Gated Channel

Molecules required to transduce light zeitgeber information to the clock have been well studied [59]–[64], although the molecular mechanisms and circuits that transduce temperature signals are less well understood. In *C. elegans*, the *tax-2* subunit of a cyclic nucleotide-gated channel has been shown to be required for responses to temperature as well as to short-wavelength visible and ultraviolet light [42]–[44],[65],[66]. We found that temperature-entrained rhythmic expression of *nlp-36*p*::gfp* expression in the intestine was significantly affected both during entrainment and free-running conditions in *tax-2* mutants (Figure 10B). Similarly, light-entrained circadian transcription of three examined genes, as measured by qRT-PCR, was also abolished in *tax-2* mutants (Figures 10C), indicating that *tax-2* is required for transduction of both light and temperature zeitgebers to the circadian clock(s). However, light-entrained cycling was unaffected in animals mutant for the transmembrane LITE-1 light receptor protein also shown to be required for light responses in *C. elegans* (Figure 10D) [42],[44],[66].

*tax-2* is expressed in several sensory neuron types, including AFD thermosensory neurons, which is the primary thermosensory neuron type in *C. elegans* [67],[68]. We found that *nlp-36*p*::gfp* expression continued to cycle, albeit with markedly reduced amplitude, in animals in which the AFD neurons had been genetically ablated (Figure 10A; gift of Miriam Goodman, Stanford University). This result suggests that the AFD neurons are not the sole source of temperature information for the clock and that one or more *tax-2*-expressing sensory neurons are required to sense and transduce environmental temperature information to entrain rhythms.

## Discussion

In the present study, we report the first identification, to our knowledge, of circadian-regulated transcripts in *C. elegans*. These transcripts are entrained by LD or WC cycles and continue to oscillate during free-running conditions with temperature-compensated periods and phases that are dependent on entraining conditions. Both light and temperature can act as zeitgebers, and they drive as well as entrain transcripts at the genome-wide level. Genome-wide circadian expression is regulated by environmental information received by a small set of sensory neuron(s), suggesting that, similar to observations in vertebrates, light and temperature information may act cell-nonautonomously to entrain the clock(s) in *C. elegans*. Our findings also imply that *C. elegans* may utilize a timekeeping mechanism that is distinct from that described previously in other animals.

In *C. elegans*, as well as in *Drosophila*, temperature cycles drive the expression of a larger set of genes than do light cycles [41],[47]. Genes implicated in metabolism appear to be enriched in the temperature-driven transcript sets, whereas genes implicated in circadian behaviors such as locomotion and reproduction are enriched in the entrained datasets in both organisms ([47] and this study). However, there is a surprising lack of overlap between the temperature- and light-entrained datasets in *C. elegans*. In contrast, in *Drosophila*, light and temperature cooperatively entrain a single transcriptional clock [47],.

What does this almost complete lack of overlap between the two *C. elegans* entrained datasets imply? At one extreme, light and temperature might act via different mechanisms to entrain two distinct clocks. Multiple clocks within a single organism are not unprecedented [4], although there is no evidence for a similar situation in metazoans. On the contrary, *Drosophila* has only a single clock that runs in multiple cells and tissues [47],[69]. Similarly, *C. elegans* may have a single clock that is entrained by light or temperature in different tissues. In this scenario, the different locations may have completely distinct sets of output genes, accounting for the nonoverlapping datasets. However, in this case, cycling clock mRNAs are expected to be shared in common between the two sets since in all eukaryotic circadian systems, from plants to mammals, many clock mRNAs undergo circadian oscillations at the transcriptional level [23],[24],[71],[72]. Since it is unlikely that a clock based on transcriptional feedback loops would have only one cycling mRNA (e.g., the *swd-3.3* gene), *C. elegans* may have a novel clock mechanism. Consistent with this hypothesis, homologs of animal clock genes do not cycle in *C. elegans*. Recent analysis has suggested that the ascidian *Ciona intestinalis* also has a divergent circadian clock, further suggesting multiple evolutionary origins of the animal circadian mechanism [73]. However, it remains possible that cycling clock mRNAs were not identified in our genome-wide studies because of masking by noncycling expression in other tissue types, or that clock proteins exhibit functional rhythmicity in *C. elegans* via post-translational modifications [74]–[76].

An alternative possibility, consistent with the presence of a single clock, is that cycling clock mRNAs as well as common output genes are present in the two driven datasets. These datasets have a statistically significant overlap, which is expected for clock and clock-regulated mRNAs. However, these transcripts do not appear to maintain cycling in free-running conditions. Although free-running rhythms are considered to be the sine qua non for circadian genes, some bona fide circadian rhythms damp quickly in constant conditions. For example, *Drosophila* peripheral rhythms damp rapidly in the absence of a light–dark cycle, including transcriptional rhythms of the clock genes themselves [77],[78]. Consistent with this scenario, we identified one of seven examined transcripts in the WC-driven datasets with characteristics of an entrained rather than a strictly driven transcript. The number of entrained transcripts is, therefore, likely to be larger, and both clock mRNAs and shared output genes may be present within the set of entrained genes whose expression is severely dampened under constant conditions. Further molecular and genetic analyses will allow us to distinguish among the above possibilities.

What are the behavioral consequences of circadian rhythmicity in *C. elegans*? Although locomotion has been reported to be under circadian control and genes implicated in locomotor behaviors are enriched in the WC-entrained dataset, this behavioral output does not appear to be particularly robust, and exhibits marked animal-to-animal variability [16],[19]. Locomotor behaviors in *C. elegans* are complex and can be dissected into multiple behavioral components [79]–[83]. Reported analyses of circadian locomotor behavior relied largely on quantification of gross overall movement [16],[18], suggesting that detailed analyses of the underlying components may reveal more robust clock control. Since reproduction-related genes are also enriched in the entrained datasets, additional biologically relevant behaviors such as egg-laying or mating may also be regulated in a circadian manner in *C. elegans*. Identification of circadian transcriptional rhythms in *C. elegans* now provides the necessary reagents to characterize the molecular identity, the neuronal circuits, and the behavioral consequences of clock function in this important model organism.

## Materials and Methods

### *C. elegans* Strains and Culture Conditions

The wild-type strain used was *C. elegans* variety Bristol, strain N2. Animals were cultured on *Escherichia coli* HB101. Mutant strains used were the following: PR671 *tax-2(p671)* [65], KG1180 *lite-1(ce314)* [66], and GN112 (*pgIs2*) (gift of M. Goodman, Stanford University). The AFD neurons are ablated in the GN112 strain via expression of reconstituted caspases under the AFD-specific *gcy-8* promoter [84],[85]. Double mutant strains were constructed using standard methods.

### Entrainment Methods

Growth-synchronized populations of wild-type late L1 larvae were transferred to plates seeded with bacteria. Two populations of animals were entrained to opposing light–dark or temperature cycles in two different incubators: 12 h at 25°C followed by 12 h at 15°C (WC), or to 12 h of light followed by 12 h of dark (LD). After reaching adulthood, the two populations of animals were transferred to the same incubator and allowed to free-run at constant 15°C (CC) or in constant darkness (DD). The time point 0 h indicates lights on or the start of the warm phase, 12 h indicates lights off or the start of the cold phase. Temperature cycling experiments were conducted in DD in temperature programmable incubators (Tritech Research). Light–dark cycling experiments were conducted at 18°C in programmable incubators (Precision Scientific; cold white light [light source F20T12/CW] at about 500–1,000 lux). To inhibit progeny production during entrainment, L4 larvae were quickly transferred to plates containing bacteria and 25 µM FUDR. Adults were harvested and washed in cold 1× M9 buffer (<15 min) every 4 h during the last day of entrainment and subsequent free-running days.

### Microarray Hybridization

Trizol Reagent (Invitrogen) was added to harvested worm samples and pellets were immediately frozen in liquid nitrogen. Extracted total RNA was treated with DNAase (New England Biolabs) as described [86]. cDNA synthesis was performed using T7-oligo(dT) primer and Superscript II (Affymetrix). Biotin-labeled cRNA was generated using the GeneChip IVT labeling kit (Affymetrix) and following the methods described in the Affymetrix GeneChip manual. Hybridization, washing, staining, and scanning of the cRNAs to the Affymetrix *C. elegans* Genome Arrays were performed as recommended by the manufacturer.

### Microarray Data Analysis

#### Expression, normalization, and standardization

The Affymetrix software was used to scan and generate .DAT and .CEL files of microarrays. Raw data can be accessed in Gene Expression Omnibus (www.ncbi.nlm.nih.gov/geo/; accession number GSE23528). The GeneChip Robust Multiarray Averaging method in the R v2.7.1 software package was used to derive log_2_ expression values for each probe set from Affymetrix-generated .CEL files. Expression values were normalized and standardized as described [87]. Briefly, for each time point in a time series experiment, the expression level relative to the mean (in log_2_ expression values) over that experiment was calculated. This normalization was performed separately for each independent time series experiment. Arrays in the independent time course experiments were standardized by setting the mean expression of each array to 0 and the variance to 1 [87]. Transcripts with low average signal intensities were excluded from further analyses.

#### ANOVA prescreening

Normalized and standardized expression values from each independent time series experiment were prescreened with ANOVA using a custom-written MATLAB code [87] (generously provided by K. Keegan). For each transcript on the arrays, a single-factor ANOVA was performed across all six time points during the entrainment or the free-running days, where each time point is a group and each array is an individual within the group. An ANOVA *p*-value of less than 0.05 was required for a transcript to pass prescreening.

#### Fourier analysis

Circadian oscillatory transcripts with 24-h periods were identified by Fourier transformation. Normalized and standardized data (see above) for each independent time series experiment were appended, and the 24-h spectral power (*F*_24_) was determined for each transcript. The spectral power score and significance of this power (power *p*-value) generated via comparison with a randomly permuted dataset (1,000 permutations) for all analyzed periods were obtained using a custom-written MATLAB code [87] (provided by K. Keegan). We elected to use a single ordering of the days in the appended profile, since these analyses (with the exception of the AC_24_ score, see below) are insensitive to the order of the days in the appended time series experiments. Transcripts that exhibited *pF*_24_-values of less than 0.02 were analyzed further.

#### Autocorrelation

An autocorrelation (AC_24_) method was used to measure the correlation between time points that are 24 h apart by fitting the six time points collected during the entrainment day to the six time points collected during the free-running day. A previously described R code [46] was used to calculate AC_24_ scores. Transcripts that passed this analysis were required to have an average AC_24_ score determined from three independent 2-d time series experiments of greater than zero.

#### False discovery rate analysis

To estimate the false positive discovery rate, the expression values of time series experiments for all datasets were randomly permuted (see Table S1). These randomly permuted data were used to perform ANOVA and *F*_24_ analysis as described above. The percentage of false discovery rate in Table S1 represents the percentage of unpermuted transcripts that were identified in the randomly permuted dataset.

#### Fold change

Transcripts exhibiting an average fold change of greater than 1.5 (log_2_-transformed expression values) between their highest and lowest expression values among the six time points of a time series experiment were selected.

### QQ Plots

QQ plots were generated to determine whether particular periods are enriched in the microarray datasets [41]. Briefly, 1,000 permutations of the ordering of time points for all transcripts within each appended time series experiment (LD only, DD only, LD/DD, WC only, CC only, and WC/CC datasets) were conducted, and the permuted Fourier score for the resulting time series experiments was calculated. The 1,000 permuted Fourier scores were then divided into a number of quantiles equal to the number represented in the unpermuted dataset. The distributions of Fourier scores found in unpermuted data and the permuted data quantiles were then compared and shown in QQ plots.

### Gene Functional Annotation Analysis

Candidate-driven and circadian-entrained transcripts were grouped by their Biological Process 3 GO term and analyzed for enrichment relative to all transcripts present on the GeneChip using functional annotation tools in DAVID (http://david.abcc.ncifcrf.gov/).

### qRT-PCR

cDNA synthesis was performed from total RNA using T7-oligo(dT) primer and Superscript III (Invitrogen). Real-time qRT-PCR analysis was performed using a Corbett Research Rotor-Gene 3000 thermal cycler. Normalization was carried out using the *act-3* actin or the *ard-1* short-chain alcohol dehydrogenase genes (expression levels of these genes do not appear to cycle). Primer sequences are available upon request. *pF*_24_-values were assessed as described above.

### In Vivo Monitoring of *gfp-pest* Expression

About 3.5-kb upstream sequences of the *nlp-36* predicted neuropeptide gene were fused to *gfp*-encoding sequences that included a PEST domain [58]. Multiple copies of the *nlp-36*p*::gfp-pest* transgene were stably integrated into the genome of wild-type animals together with the *unc-122*p*::dsRed* marker to generate the PY7644 (*oyIs80*) strain. To obtain a growth-synchronized population of L1 larvae, ten adult animals were transferred to seeded plates and were allowed to lay approximately 50 eggs. Wild-type animals, *tax-2* mutants, and AFD-killed L1 larvae carrying the *oyIs80* transgene were entrained for 4 d to temperature cycles (12 h∶12 h) under well-fed conditions, and intestinal *nlp-36*p*::gfp-pest* fluorescence intensity was determined at specific time points during entrainment and free-running days. No FUDR was added during the entrainment protocol. Images of 15–20 adult animals were acquired at 100× magnification using a compound microscope (Zeiss) equipped with epifluorescence and a CCD camera (Hamamatsu Photonics) at each time point. Levels of intestinal expression were quantified in arbitrary units using Image J (National Institutes of Health).

## Supporting Information

Figure S1 **Analysis of temperature-driven transcripts.** (A) Comparison of qRT-PCR (gray) and microarray expression array (black) data of randomly selected WC-driven transcripts. The probability of significance of circadian cycling (*pF*_24_) as compared to a randomized dataset was calculated by appending each independent dataset. There are two probe sets each for *acs-2* and *cyp-35C1* on the GeneChips. Bars below the graphs denote the entrainment protocols, with red and blue bars indicating the warm (25°C) and cold (15°C) phases, respectively. (B) qRT-PCR data of temperature-driven transcripts under constant conditions (15°C). Data shown are an average of three biologically independent replicates per time point for the microarray data and two biologically independent replicates per time point for the qRT-PCR data.
(0.70 MB TIF)

Figure S2 **Analysis of light- and temperature-entrained transcripts.** (A) Comparison of qRT-PCR (gray) and microarray expression array (black) data of arbitrarily selected light- and temperature-entrained transcripts. The probability of significance of circadian cycling (*pF*_24_) as compared to a randomized dataset was calculated by appending each independent microarray or qRT-PCR time course experiment. Bars below the graphs denote the entrainment protocols, with white, black, and gray bars indicating the light, dark, and subjective light phases, respectively, and with red, blue, and light blue bars indicating the warm (25°C), cold (15°C), and subjective warm phases, respectively. Data shown are an average of three biologically independent replicates per time point for the microarray data, and two biologically independent replicates per time point for the qRT-PCR data. (B) qRT-PCR data of light- and temperature-entrained transcripts under constant conditions. Data shown are an average of two biologically independent replicates per time point for the microarray data, and two biologically independent replicates per time point for the qRT-PCR data, with the exception of *K11H12.6* and *C33F10.4*, for which data from one experiment were analyzed.
(0.80 MB TIF)

Figure S3 **Phases of transcripts from the WC-driven datasets.** Animals were entrained to the indicated *T*-cycles for 3 d and RNA was collected on the fourth day. qRT-PCR data for each time point are the average of two technical replicates from one biological experiment.
(0.84 MB TIF)

Figure S4 ***nlp-36*** **p** ***::gfp*** **expression does not cycle upon light entrainment.** Fluorescence intensities in wild-type animals (gray bars) or *tax-2* mutant animals (red bars) carrying the *nlp-36*p*::gfp* transgene entrained to light cycles (LD/DD). Error bars indicate the standard error of the mean (s.e.m). Data shown are from two independent experiments.
(0.18 MB TIF)

Table S1 **False discovery rate analysis of datasets.**
(0.11 MB DOC)

Table S2 **GO categories and lists of all light- and temperature-driven transcripts.**
(1.18 MB XLS)

Table S3 **GO categories and lists of all light- and temperature-entrained transcripts.**
(0.47 MB XLS)

Table S4 **Fourier analyses of periods at different temperatures.**
(0.06 MB DOC)

Table S5 **Transcripts of** ***C. elegans*** **clock genes do not oscillate in a circadian manner.**
(0.06 MB DOC)

## Abbreviations

CC: constant cold
CW: cold/warm
DD: constant darkness
FUDR: 5-fluoro-2-deoxyuridine
*gfp*: *green fluorescent protein*
GO: Gene Ontology
LD: light/dark
QQ: quantile–quantile
qRT-PCR: quantitative reverse transcription PCR
s.e.m.: standard error of the mean
WC: warm/cold

## References

[1] Young M. W, Kay S. A (2001). Time zones: a comparative genetics of circadian clocks.. Nat Rev Genet.

[2] Rosbash M (2009). The implications of multiple circadian clock origins.. PLoS Biol.

[3] Kondo T (2007). A cyanobacterial circadian clock based on the Kai oscillator.. Cold Spring Harb Symp Quant Biol.

[4] Bell-Pedersen D, Cassone V. M, Earnest D. J, Golden S. S, Hardin P. E (2005). Circadian rhythms from multiple oscillators: lessons from diverse organisms.. Nat Rev Genet.

[5] Yu W, Hardin P. E (2006). Circadian oscillators of *Drosophila* and mammals.. J Cell Sci.

[6] Johnson C. H, Mori T, Xu Y (2008). A cyanobacterial circadian clockwork.. Curr Biol.

[7] Allada R, Emery P, Takahashi J. S, Rosbash M (2001). Stopping time: the genetics of fly and mouse circadian clocks.. Annu Rev Neurosci.

[8] Allada R, Chung B. Y (2010). Circadian organization of behavior and physiology in *Drosophila*.. Annu Rev Physiol.

[9] de Bono M, Maricq A. V (2005). Neuronal substrates of complex behaviors in *C. elegans*.. Annu Rev Neurosci.

[10] Fielenbach N, Antebi A (2008). *C. elegans* dauer formation and the molecular basis of plasticity.. Genes Dev.

[11] Antebi A (2007). Genetics of aging in *Caenorhabditis elegans*.. PLoS Genet.

[12] Conradt B (2009). Genetic control of programmed cell death during animal development.. Annu Rev Genet.

[13] Ramot D, MacInnis B. L, Lee H. C, Goodman M. B (2008). Thermotaxis is a robust mechanism for thermoregulation in *Caenorhabditis elegans* nematodes.. J Neurosci.

[14] Robinson A. F (1994). Movement of five nematode species through sand subjected to natural temperature gradient fluctuations.. J Nematol.

[15] Kiontke K, Sudhaus W (2006). Ecology of *Caenorhabditis* species.. WormBook.

[16] Saigusa T, Ishizaki S, Watabiki S, Ishii N, Tanakadate A (2002). Circadian behavioural rhythm in *Caenorhabditis elegans*.. Curr Biol.

[17] Kippert F, Saunders D. S, Blaxter M. L (2002). *Caenorhabditis elegans* has a circadian clock.. Curr Biol.

[18] Simonetta S. H, Golombek D. A (2007). An automated tracking system for *Caenorhabditis elegans* locomotor behavior and circadian studies application.. J Neurosci Methods.

[19] Simonetta S. H, Migliori M. L, Romanowski A, Golombek D. A (2009). Timing of locomotor activity circadian rhythms in *Caenorhabditis elegans*.. PLoS ONE.

[20] Simonetta S. H, Romanowski A, Minniti A. N, Inestrosa N. C, Golombek D. A (2008). Circadian stress tolerance in adult *Caenorhabditis elegans*.. J Comp Physiol A Neuroethol Sens Neural Behav Physiol.

[21] Jeon M, Gardner H. F, Miller E. A, Deshler J, Rougvie A. E (1999). Similarity of the *C. elegans* developmental timing protein LIN-42 to circadian rhythm proteins.. Science.

[22] McDonald M. J, Rosbash M, Emery P (2001). Wild-type circadian rhythmicity is dependent on closely spaced E boxes in the *Drosophila timeless* promoter.. Mol Cell Biol.

[23] Hardin P. E, Hall J. C, Rosbash M (1992). Circadian oscillations in *period* gene mRNA levels are transcriptionally regulated.. Proc Natl Acad Sci U S A.

[24] Sehgal A, Rothenfluh-Hilfiker A, Hunter-Ensor M, Chen Y, Myers M. P (1995). Rhythmic expression of timeless: a basis for promoting circadian cycles in period gene autoregulation.. Science.

[25] Tei H, Okamura H, Shigeyoshi Y, Fukuhara C, Ozawa R (1997). Circadian oscillation of a mammalian homologue of the *Drosophila period* gene.. Nature.

[26] Shearman L. P, Zylka M. J, Weaver D. R, Kolakowski L. F, Reppert S. M (1997). Two period homologs: circadian expression and photic regulation in the suprachiasmatic nuclei.. Neuron.

[27] Gallego M, Virshup D. M (2007). Post-translational modifications regulate the ticking of the circadian clock.. Nat Rev Mol Cell Biol.

[28] Mehra A, Baker C. L, Loros J. J, Dunlap J. C (2009). Post-translational modifications in circadian rhythms.. Trends Biochem Sci.

[29] Liu Y, Garceau N. Y, Loros J. J, Dunlap J. C (1997). Thermally regulated translational control of FRQ mediates aspects of temperature responses in the *Neurospora* circadian clock.. Cell.

[30] Garceau N. Y, Liu Y, Loros J. J, Dunlap J. C (1997). Alternative initiation of translation and time-specific phosphorylation yield multiple forms of the essential clock protein FREQUENCY.. Cell.

[31] Nakajima M, Imai K, Ito H, Nishiwaki T, Murayama Y (2005). Reconstitution of circadian oscillation of cyanobacterial KaiC phosphorylation in vitro.. Science.

[32] Kadener S, Menet J. S, Sugino K, Horwich M. D, Weissbein U (2009). A role for microRNAs in the *Drosophila* circadian clock.. Genes Dev.

[33] Claridge-Chang A, Wijnen H, Naef F, Boothroyd C, Rajewsky N (2001). Circadian regulation of gene expression systems in the *Drosophila* head.. Neuron.

[34] Ceriani M. F, Hogenesch J. B, Yanovsky M, Panda S, Straume M (2002). Genome-wide expression analysis in *Drosophila* reveals genes controlling circadian behavior.. J Neurosci.

[35] McDonald M. J, Rosbash M (2001). Microarray analysis and organization of circadian gene expression in *Drosophila*.. Cell.

[36] Ueda H. R, Matsumoto A, Kawamura M, Iino M, Tanimura T (2002). Genome-wide transcriptional orchestration of circadian rhythms in *Drosophila*.. J Biol Chem.

[37] Akhtar R. A, Reddy A. B, Maywood E. S, Clayton J. D, King V. M (2002). Circadian cycling of the mouse liver transcriptome, as revealed by cDNA microarray, is driven by the suprachiasmatic nucleus.. Curr Biol.

[38] Correa A, Lewis Z. A, Greene A. V, March I. J, Gomer R. H (2003). Multiple oscillators regulate circadian gene expression in *Neurospora*.. Proc Natl Acad Sci U S A.

[39] Duffield G. E, Best J. D, Meurers B. H, Bittner A, Loros J. J (2002). Circadian programs of transcriptional activation, signaling, and protein turnover revealed by microarray analysis of mammalian cells.. Curr Biol.

[40] Nowrousian M, Duffield G. E, Loros J. J, Dunlap J. C (2003). The *frequency* gene is required for temperature-dependent regulation of many clock-controlled genes in *Neurospora crassa*.. Genetics.

[41] Wijnen H, Naef F, Boothroyd C, Claridge-Chang A, Young M. W (2006). Control of daily transcript oscillations in *Drosophila* by light and the circadian clock.. PLoS Genet.

[42] Ward A, Liu J, Feng Z, Xu X. Z (2008). Light-sensitive neurons and channels mediate phototaxis in *C. elegans*.. Nat Neurosci.

[43] Hedgecock E. M, Russell R. L (1975). Normal and mutant thermotaxis in the nematode *Caenorhabditis elegans*.. Proc Natl Acad Sci U S A.

[44] Liu J, Ward A, Gao J, Dong Y, Nishio N (2010). *C. elegans* phototransduction requires a G protein-dependent cGMP pathway and a taste receptor homolog.. Nat Neurosci.

[45] Mitchell D. H, Stiles J. W, Santelli J, Sanadi D. R (1979). Synchronous growth and aging of *Caenorhabditis elegans* in the presence of fluorodeoxyuridine.. J Gerontol.

[46] Wijnen H, Naef F, Young M. W (2005). Molecular and statistical tools for circadian transcript profiling.. Methods Enzymol.

[47] Boothroyd C. E, Wijnen H, Naef F, Saez L, Young M. W (2007). Integration of light and temperature in the regulation of circadian gene expression in *Drosophila*.. PLoS Genet.

[48] Hughes M. E, DiTacchio L, Hayes K. R, Vollmers C, Pulivarthy S (2009). Harmonics of circadian gene transcription in mammals.. PLoS Genet.

[49] Ashburner M, Ball C. A, Blake J. A, Botstein D, Butler H (2000). Gene ontology: tool for the unification of biology. The Gene Ontology Consortium.. Nat Genet.

[50] Hardin P. E (2006). Essential and expendable features of the circadian timekeeping mechanism.. Curr Opin Neurobiol.

[51] Hastings J. W, Sweeney B. M (1957). On the mechanism of temperature independence in a biological clock.. Proc Natl Acad Sci U S A.

[52] Roenneberg T, Dragovic Z, Merrow M (2005). Demasking biological oscillators: properties and principles of entrainment exemplified by the *Neurospora* circadian clock.. Proc Natl Acad Sci U S A.

[53] Merrow M, Brunner M, Roenneberg T (1999). Assignment of circadian function for the *Neurospora* clock gene *frequency*.. Nature.

[54] Aschoff J, Pohl H (1978). Phase relations between a circadian rhythm and its zeitgeber within the range of entrainment.. Naturwissenschaften.

[55] Roenneberg T, Daan S, Merrow M (2003). The art of entrainment.. J Biol Rhythms.

[56] Brown S. A, Ripperger J, Kadener S, Fleury-Olela F, Vilbois F (2005). PERIOD1-associated proteins modulate the negative limb of the mammalian circadian oscillator.. Science.

[57] Kloss B, Price J. L, Saez L, Blau J, Rothenfluh A (1998). The *Drosophila* clock gene *double-time* encodes a protein closely related to human casein kinase Iepsilon.. Cell.

[58] Frand A. R, Russel S, Ruvkun G (2005). Functional genomic analysis of *C. elegans* molting.. PLoS Biol.

[59] Berson D. M, Dunn F. A, Takao M (2002). Phototransduction by retinal ganglion cells that set the circadian clock.. Science.

[60] Hattar S, Lucas R. J, Mrosovsky N, Thompson S, Douglas R. H (2003). Melanopsin and rod-cone photoreceptive systems account for all major accessory visual functions in mice.. Nature.

[61] Stanewsky R, Kaneko M, Emery P, Beretta B, Wager-Smith K (1998). The *cryb* mutation identifies cryptochrome as a circadian photoreceptor in *Drosophila*.. Cell.

[62] Emery P, So W. V, Kaneko M, Hall J. C, Rosbash M (1998). CRY, a *Drosophila* clock and light-regulated cryptochrome, is a major contributor to circadian rhythm resetting and photosensitivity.. Cell.

[63] van der Horst G. T, Muijtjens M, Kobayashi K, Takano R, Kanno S (1999). Mammalian Cry1 and Cry2 are essential for maintenance of circadian rhythms.. Nature.

[64] Ballario P, Vittorioso P, Magrelli A, Talora C, Cabibbo A (1996). White collar-1, a central regulator of blue light responses in *Neurospora*, is a zinc finger protein.. EMBO J.

[65] Coburn C, Bargmann C. I (1996). A putative cyclic nucleotide-gated channel is required for sensory development and function in *C. elegans*.. Neuron.

[66] Edwards S. L, Charlie N. K, Milfort M. C, Brown B. S, Gravlin C. N (2008). A novel molecular solution for ultraviolet light detection in *Caenorhabditis elegans*.. PLoS Biol.

[67] Prahlad V, Cornelius T, Morimoto R. I (2008). Regulation of the cellular heat shock response in *Caenorhabditis elegans* by thermosensory neurons.. Science.

[68] Mori I, Ohshima Y (1995). Neural regulation of thermotaxis in *Caenorhabditis elegans*.. Nature.

[69] Yoshii T, Vanin S, Costa R, Helfrich-Forster C (2009). Synergic entrainment of *Drosophila*'s circadian clock by light and temperature.. J Biol Rhythms.

[70] Matsumoto A, Matsumoto N, Harui Y, Sakamoto M, Tomioka K (1998). Light and temperature cooperate to regulate the circadian locomotor rhythm of wild type and *period* mutants of *Drosophila* melanogaster.. J Insect Physiol.

[71] Gekakis N, Staknis D, Nguyen H. B, Davis F. C, Wilsbacher L. D (1998). Role of the CLOCK protein in the mammalian circadian mechanism.. Science.

[72] Alabadi D, Oyama T, Yanovsky M. J, Harmon F. G, Mas P (2001). Reciprocal regulation between TOC1 and LHY/CCA1 within the *Arabidopsis* circadian clock.. Science.

[73] Minamoto T, Hanai S, Kadota K, Oishi K, Matsumae H (2009). Circadian clock in *Ciona intestinalis* revealed by microarray analysis and oxygen consumption.. J Biochem.

[74] Cha J, Huang G, Guo J, Liu Y (2007). Posttranslational control of the *Neurospora* circadian clock.. Cold Spring Harb Symp Quant Biol.

[75] Zheng X, Sehgal A (2008). Probing the relative importance of molecular oscillations in the circadian clock.. Genetics.

[76] Harms E, Kivimae S, Young M. W, Saez L (2004). Posttranscriptional and posttranslational regulation of clock genes.. J Biol Rhythms.

[77] Hardin P. E (1994). Analysis of period mRNA cycling in *Drosophila* head and body tissues indicates that body oscillators behave differently from head oscillators.. Mol Cell Biol.

[78] Plautz J. D, Kaneko M, Hall J. C, Kay S. A (1997). Independent photoreceptive circadian clocks throughout *Drosophila*.. Science.

[79] Chalfie M, Sulston J. E, White J. G, Southgate E, Thomson J. N (1985). The neural circuit for touch sensitivity in *Caenorhabditis elegans*.. J Neurosci.

[80] Croll N. A (1975). Components and patterns in the behavior of the nematode *Caenorhabditis elegans*.. J Zool (1987).

[81] Gray J. M, Hill J. J, Bargmann C. I (2005). A circuit for navigation in *Caenorhabditis elegans*.. Proc Natl Acad Sci U S A.

[82] Pierce-Shimomura J. T, Morse T. M, Lockery S. R (1999). The fundamental role of pirouettes in *Caenorhabditis elegans* chemotaxis.. J Neurosci.

[83] Srivastava N, Clark D. A, Samuel A. D (2009). Temporal analysis of stochastic turning behavior of swimming *C. elegans*.. J Neurophysiol.

[84] Yu S, Avery L, Baude E, Garbers D. A (1997). Guanylyl cyclase expression in specific sensory neurons: A new family of chemosensory receptors.. Proc Natl Acad Sci U S A.

[85] Chelur D. S, Chalfie M (2007). Targeted cell killing by reconstituted caspases.. Proc Natl Acad Sci U S A.

[86] Portman D. S, The *C. elegans* Research Community, editor (2006). Profiling *C. elegans* gene expression with DNA microarrays. WormBook.

[87] Keegan K. P, Pradhan S, Wang J. P, Allada R (2007). Meta-analysis of *Drosophila* circadian microarray studies identifies a novel set of rhythmically expressed genes.. PLoS Comput Biol.

