# Supplementary figures and images for: Genome-Wide Analysis of Light- and Temperature-Entrained Circadian Transcripts in Caenorhabditis elegans
Source: PLoS Biol. 2010 Oct 12;8(10):e1000503. doi: 10.1371/journal.pbio.1000503 (PMC2953524; doi:10.1371/journal.pbio.1000503)

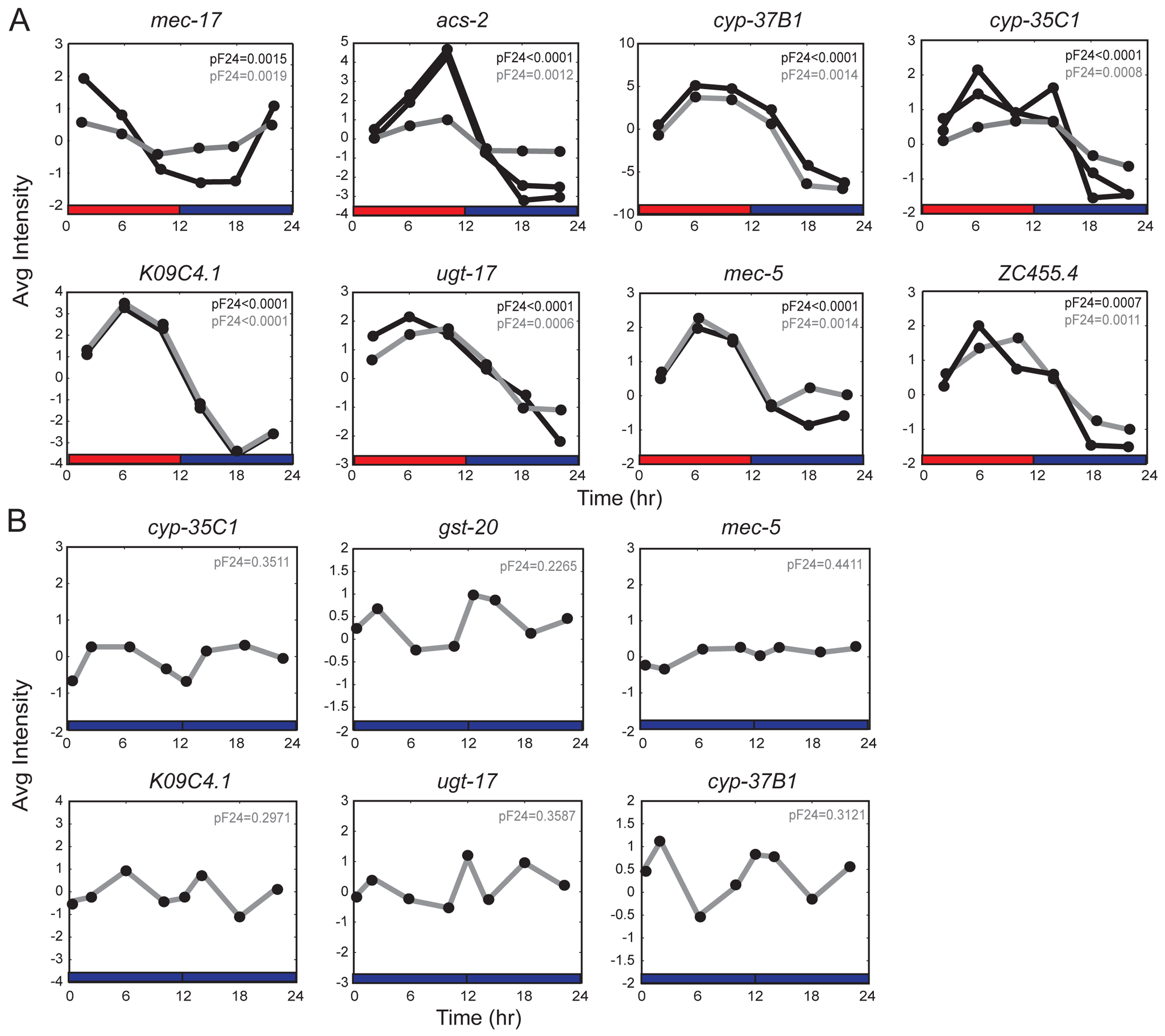

Supplement: Figure S1 — Analysis of temperature-driven transcripts. (A) Comparison of qRT-PCR (gray) and microarray expression array (black) data of randomly selected WC-driven transcripts. The probability of significance of circadian cycling (pF 24) as compared to a randomized dataset was calculated by appending each independent dataset. There are two probe sets each for acs-2 and cyp-35C1 on the GeneChips. Bars below the graphs denote the entrainment protocols, with red and blue bars indicating the warm (25°C) and cold (15°C) phases, respectively. (B) qRT-PCR data of temperature-driven transcripts under constant conditions (15°C). Data shown are an average of three biologically independent replicates per time point for the microarray data and two biologically independent replicates per time point for the qRT-PCR data. (0.70 MB TIF) [file pbio.1000503.s001.tif]

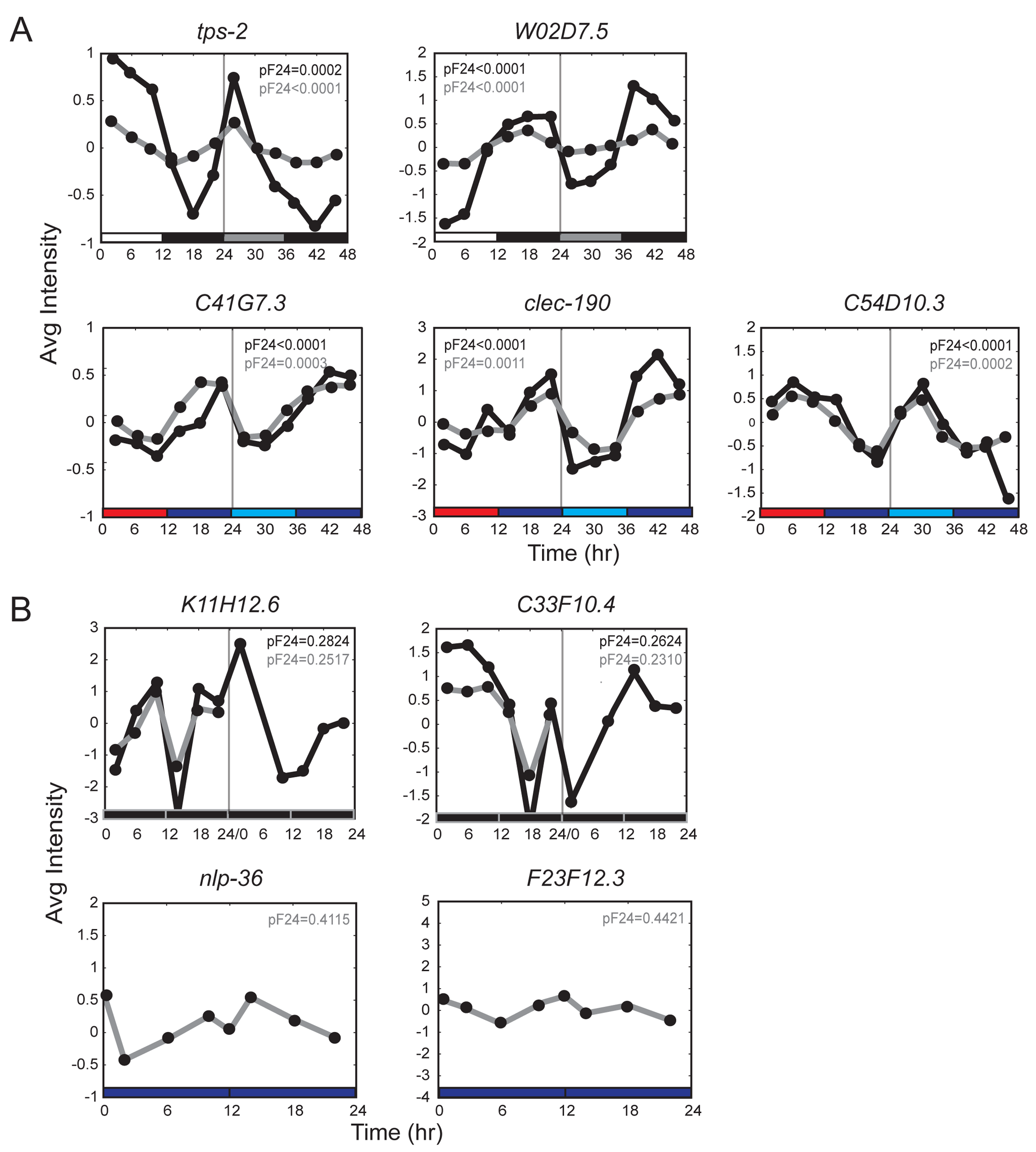

Supplement: Figure S2 — Analysis of light- and temperature-entrained transcripts. (A) Comparison of qRT-PCR (gray) and microarray expression array (black) data of arbitrarily selected light- and temperature-entrained transcripts. The probability of significance of circadian cycling (pF 24) as compared to a randomized dataset was calculated by appending each independent microarray or qRT-PCR time course experiment. Bars below the graphs denote the entrainment protocols, with white, black, and gray bars indicating the light, dark, and subjective light phases, respectively, and with red, blue, and light blue bars indicating the warm (25°C), cold (15°C), and subjective warm phases, respectively. Data shown are an average of three biologically independent replicates per time point for the microarray data, and two biologically independent replicates per time point for the qRT-PCR data. (B) qRT-PCR data of light- and temperature-entrained transcripts under constant conditions. Data shown are an average of two biologically independent replicates per time point for the microarray data, and two biologically independent replicates per time point for the qRT-PCR data, with the exception of K11H12.6 and C33F10.4, for which data from one experiment were analyzed. (0.80 MB TIF) [file pbio.1000503.s002.tif]

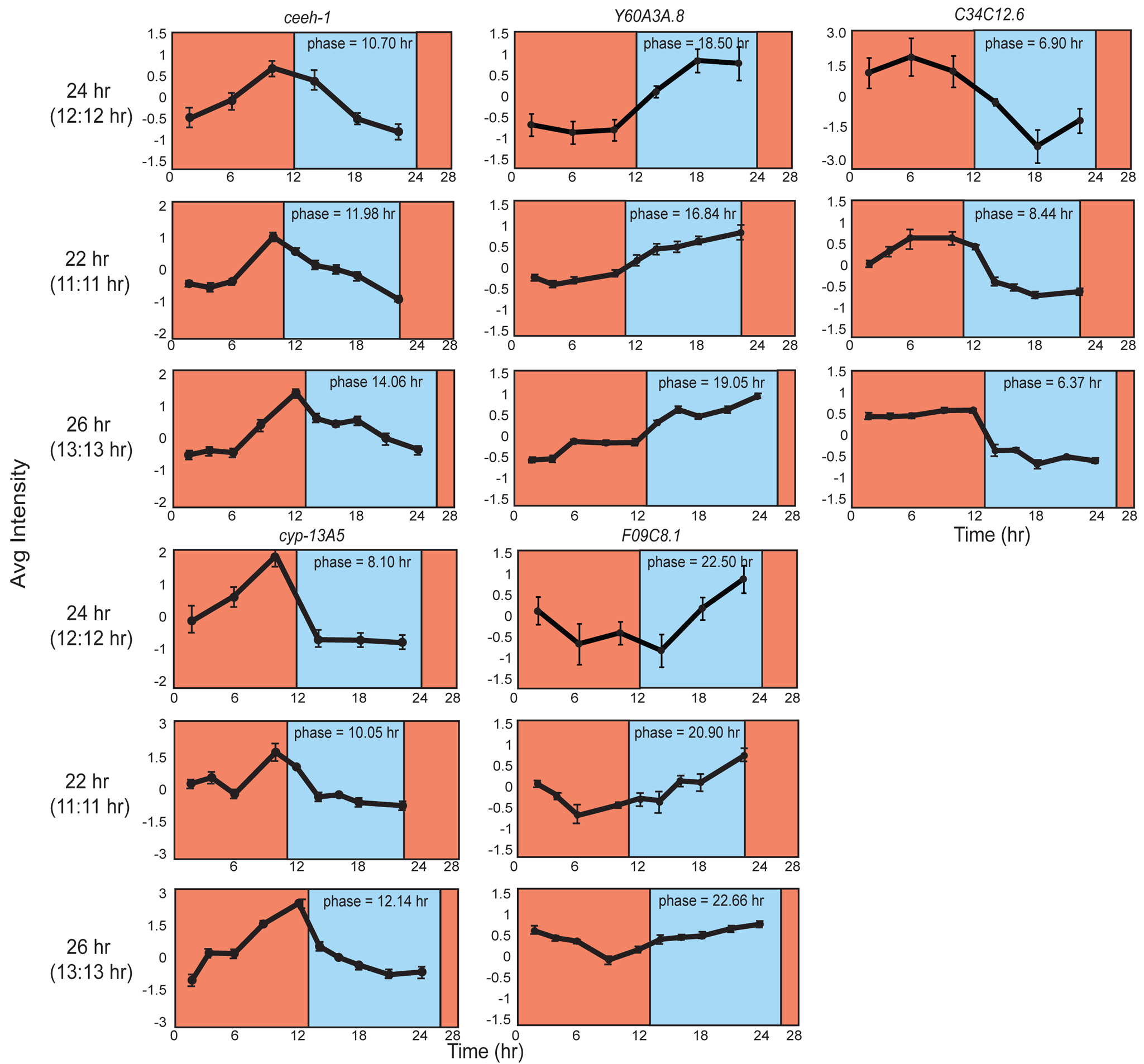

Supplement: Figure S3 — Phases of transcripts from the WC-driven datasets. Animals were entrained to the indicated T-cycles for 3 d and RNA was collected on the fourth day. qRT-PCR data for each time point are the average of two technical replicates from one biological experiment. (0.84 MB TIF) [file pbio.1000503.s003.tif]

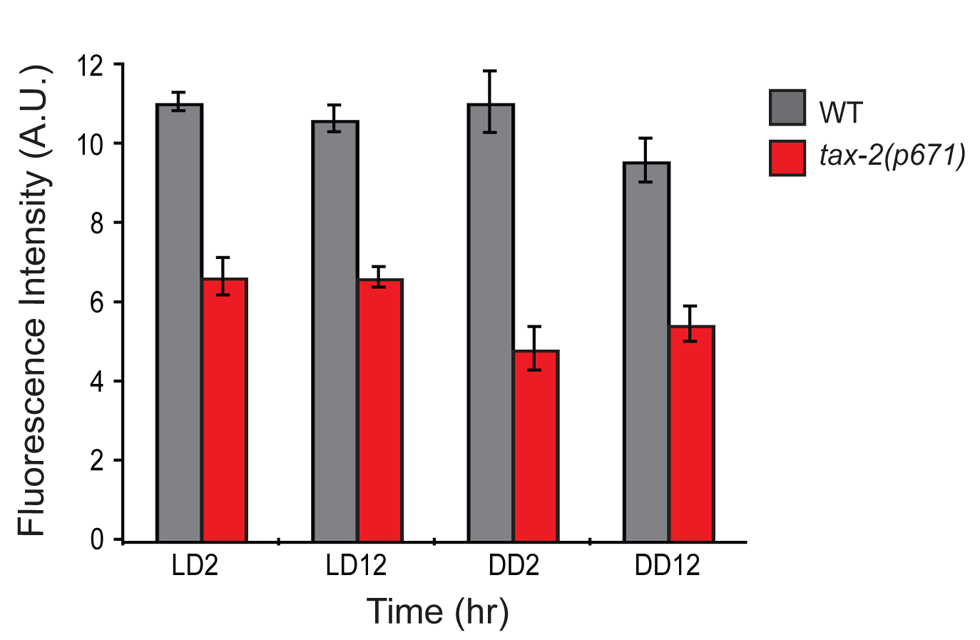

Supplement: Figure S4 — nlp-36 p ::gfp expression does not cycle upon light entrainment. Fluorescence intensities in wild-type animals (gray bars) or tax-2 mutant animals (red bars) carrying the nlp-36p::gfp transgene entrained to light cycles (LD/DD). Error bars indicate the standard error of the mean (s.e.m). Data shown are from two independent experiments. (0.18 MB TIF) [file pbio.1000503.s004.tif]
