# Supplementary material for: Genome-Wide Analysis of Light- and Temperature-Entrained Circadian Transcripts in Caenorhabditis elegans
Source: PLoS Biol. 2010 Oct 12;8(10):e1000503. doi: 10.1371/journal.pbio.1000503 (PMC2953524; doi:10.1371/journal.pbio.1000503)
Supplement: Table S1 — False discovery rate analysis of datasets. (0.11 MB DOC) [file pbio.1000503.s005.doc]

**Supplemental Table 1.** FDR analysis of data sets.

| **Dataset** | **Days** | **Analysis** | **Number of Probe sets**^1^ | | **FDR**  **%** |
| --- | --- | --- | --- | --- | --- |
|  |  |  | **Detected**  **Real Data** | **Detected Randomized Data**^2^ |  |
| LD/DD | 6 | Probe Sets | 13990 | 13990 | - |
|  |  | Pass pF24<0.02 | 2091 | 276 | 10 |
|  |  | Pass pANOVA(DD)<0.05 | 1024 | 634 | 62 |
|  |  | Pass pANOVA(DD) and pF24 | 618 | 67 | 11 |
|  |  | Pass pANOVA, pF24, AC24, and LD/DD-fold change | 431 | 5 | 1.1 |
| **LD/DD** |  | **Pass pANOVA, pF24, AC24, LD/DD-fold change, and AC24 control**^3^ | **294** | 0 | 0 |
| DD | 3 | Probe Sets | 14082 | 14082 | - |
|  |  | Pass pF24<0.02 | 1148 | 265 | 23 |
|  |  | Pass pANOVA<0.05 | 1026 | 637 | 62 |
|  |  | Pass pANOVA and pF24 | 587 | 11 | 2 |
|  |  | Pass pANOVA, pF24, AC24, and DD-fold change, and AC24 control**^3^** | 367 | 0 | 0 |
| **DD-only** |  | **Pass all filters/thresholds, and do not cycle in LD and LD/DD** | **112** | - | - |
| LD | 3 | Probe Sets | 13679 | 13679 | - |
|  |  | Pass pF24<0.02 | 2275 | 279 | 12 |
|  |  | Pass pANOVA<0.05 | 1778 | 631 | 35 |
|  |  | Pass pANOVA and pF24 | 1378 | 10 | 0.7 |
|  |  | Pass pANOVA, pF24, AC24, LD-fold change, and AC24 control^3^ | 913 | 1 | 0.1 |
| **LD-only** |  | **Pass all filters/thresholds, and do not cycle in DD and LD/DD** | **775** | - | - |
| WC/CC | 6 | Probe Sets | 14331 | 14331 | - |
|  |  | Pass pF24<0.02 | 1243 | 310 | 25 |
|  |  | Pass pANOVA(CC)<0.05 | 658 | 625 | 95 |
|  |  | Pass pANOVA(CC) and pF24 | 135 | 55 | 41 |
| **WC/CC** |  | **Pass pANOVA, pF24, AC24, and WC/CC-fold change** | **88** | 2 | 2.3 |
| CC | 3 | Probe Sets | 14146 | 14146 | - |
|  |  | Pass pF24<0.02 | 705 | 284 | 40 |
|  |  | Pass pANOVA<0.05 | 657 | 526 | 80 |
|  |  | Pass pANOVA and pF24 | 346 | 101 | 29 |
|  |  | Pass pANOVA, pF24, AC24, and CC-fold change | 326 | 8 | 2.5 |
| **CC-only** |  | **Pass all filters/thresholds, and do not cycle in WC and WC/CC** | **198** | - | - |
| WC | 5 | Probe Sets | 14624 | 14624 | - |
|  |  | Pass pF24<0.02 | 2595 | 297 | 11 |
|  |  | Pass pANOVA<0.05 | 2923 | 668 | 23 |
|  |  | Pass pANOVA and pF24 | 1948 | 153 | 7.9 |
|  |  | Pass pANOVA, pF24, AC24, and WC-fold change | 1899 | 14 | 0.73 |
| **WC-only** |  | **Pass all filters/thresholds, and do not cycle in CC and WC/CC** | **1817** | - | - |

**^1^ Transcripts with log2 transformed expressed value lower than 10 average were excluded (see Material and Methods).**

**^2^ Transcripts were randomly permuted once (see Material and Methods).**

**^3^ AC24 control was calculated by measuring the correlation between time-points that are 24 hr apart by fitting the six time points collected during the entrained day to the six time points during the non-entrained control day performed in parallel. Transcripts from two-independent one-day time-series experiments were required to have an average AC24 score of <0 (see Material and Methods).**
