# Supplementary material for: Genome-Wide Analysis of Light- and Temperature-Entrained Circadian Transcripts in Caenorhabditis elegans
Source: PLoS Biol. 2010 Oct 12;8(10):e1000503. doi: 10.1371/journal.pbio.1000503 (PMC2953524; doi:10.1371/journal.pbio.1000503)
Supplement: Table S4 — Fourier analyses of periods at different temperatures. (0.06 MB DOC) [file pbio.1000503.s008.doc]

**Supplemental Table 4.** Fourier analyses of periods at different temperatures.

| **Gene** | **T(°C)** | **Fourier analysis of zero-padded data^1^** | | | | | | | | | | | |
| --- | --- | --- | --- | --- | --- | --- | --- | --- | --- | --- | --- | --- | --- |
|  |  | **F28.8** | **pF28.80** | **F26.19** | **pF26.19** | **F24** | **pF24** | **F22.15** | **pF22.15** | **F20.57** | **pF20.57** | **F19.2** | **pF19.2** |
| *K11H12.6* | 15 | 0.04203 | 0.2237 | 0.12983 | 0.0051 | 0.16312 | 0.0014 | 0.11018 | 0.0138 | 0.04206 | 0.2288 | 0.01123 | 0.6776 |
| *K11H12.6* | 25 | 0.08223 | 0.0450 | 0.21679 | 0 | 0.22623 | 0 | 0.10676 | 0.0145 | 0.01819 | 0.5184 | 0.00101 | 0.9632 |
| *C33F10.4* | 15 | 0.01565 | 0.5630 | 0.04711 | 0.1732 | 0.10429 | 0.0124 | 0.10831 | 0.0136 | 0.04307 | 0.1936 | 0.00993 | 0.6903 |
| *C33F10.4* | 25 | 0.00728 | 0.7770 | 0.04554 | 0.1943 | 0.11307 | 0.0108 | 0.11984 | 0.0082 | 0.04957 | 0.1641 | 0.00856 | 0.7447 |

^1^Probabilities and Fourier scores of each period were calculated by zero-padding two appended biologically independent two-day time-series to 72 time points. Probability values of <0.02 are underlined.
