# Supplementary material for: Genome-Wide Analysis of Light- and Temperature-Entrained Circadian Transcripts in Caenorhabditis elegans
Source: PLoS Biol. 2010 Oct 12;8(10):e1000503. doi: 10.1371/journal.pbio.1000503 (PMC2953524; doi:10.1371/journal.pbio.1000503)
Supplement: Table S5 — Transcripts of C. elegans clock genes do not oscillate in a circadian manner. (0.06 MB DOC) [file pbio.1000503.s009.doc]

**Supplemental Table 5.** Transcripts of *C. elegans* clock genes do not oscillate in a circadian manner.

| **Probe**  **set** | **Gene** | **Homolog** | **Fourier** | | | | | | | | | | | |
| --- | --- | --- | --- | --- | --- | --- | --- | --- | --- | --- | --- | --- | --- | --- |
|  |  |  | **LD/DD**  **(6 days)** | | **DD**  **(3 days)** | | **LD**  **(3 days)** | | **WC/CC**  **(6 days)** | | **CC**  **(3 days)** | | **WC**  **(5 days)** | |
|  |  |  | **F24** | **pF24** | **F24** | **pF24** | **F24** | **pF24** | **F24** | **pF24** | **F24** | **pF24** | **F24** | **pF24** |
| 172057_x_at | *aha-1* | Clock / Cycle | 0.249 | 0.008 | 0.232 | 0.040 | 0.311 | 0.013 | 0.132 | 0.098 | 0.018 | 0.868 | 0.212 | 0.036 |
| 172967_x_at | *aha-1* | Clock / Cycle | 0.151 | 0.069 | 0.075 | 0.367 | 0.234 | 0.048 | 0.073 | 0.289 | 0.023 | 0.829 | 0.131 | 0.135 |
| 185562_s_at | *tim-1* | Timeless | 0.129 | 0.103 | 0.074 | 0.550 | 0.246 | 0.070 | 0.032 | 0.586 | 0.006 | 0.939 | 0.055 | 0.445 |
| 189943_at | *atf-2* | Vrille | 0.144 | 0.078 | 0.081 | 0.461 | 0.454 | 0.004 | 0.157 | 0.059 | 0.172 | 0.077 | 0.023 | 0.704 |
| 176596_at | *lin-42* | Period | 0.034 | 0.684 | 0.090 | 0.585 | 0.040 | 0.545 | 0.047 | 0.616 | 0.025 | 0.708 | 0.069 | 0.443 |
| 179896_at | *lin-42* | Period | 0.109 | 0.150 | 0.009 | 0.938 | 0.296 | 0.058 | 0.005 | 0.925 | 0.015 | 0.869 | 0.011 | 0.854 |
